# ADP-ribosylating adjuvant reveals plasticity in cDC1 cells that drive mucosal Th17 cell development and protection against influenza virus infection

**DOI:** 10.1038/s41385-022-00510-1

**Published:** 2022-04-13

**Authors:** Mohammad Arabpour, Cristina Lebrero-Fernandez, Karin Schön, Anneli Strömberg, Vanja Börjesson, Katharina Lahl, Marlies Ballegeer, Xavier Saelens, Davide Angeletti, William Agace, Nils Lycke

**Affiliations:** 1 MIVAC-Mucosal Immunobiology & Vaccine Center, Department of Microbiology and Immunology, Institute of Biomedicine, University of Gothenburg, Gothenburg, Sweden; 2 Bioinformatics Core Facility, The Sahlgrenska Academy, University of Gothenburg, Gothenburg, Sweden; 3 Immunology Section, Lund University, BMC D14, 221-84 Lund, Sweden; 4 VIB-UGent Center for Medical Biotechnology, VIB, Ghent, Belgium and Department of Biomedical Molecular Biology, Ghent University, Ghent, Belgium; 5 Mucosal Immunology Group, Department of Health Technology, Technical University of Denmark, Kemitorvet, 2800 Kgs, Lyngby, Denmark

## Abstract

Migratory dendritic cells expressing CD103 are the targets for mucosal vaccines. These belong to either of two lineage-restricted subsets, cDC1 or cDC2 cells, which have been linked to priming of functionally distinct CD4 T cells. However, recent studies have identified plasticity in cDC2 cells with overlapping functions with cDC1 cells, while the converse has not been reported. We genetically engineered a vaccine adjuvant platform that targeted the cholera toxin A1 (CTA1) ADP-ribosylating enzyme to CD103^+^ cDC1 and cDC2 cells using a single-chain antibody (scFv) to CD103. Unexpectedly, intranasal immunization with the CTA1-svFcCD103 adjuvant modified cDC1 cells to effectively prime Th17 cells, a function previously limited to cDC2 cells. In fact, cDC2 cells were dispensible, while cDC1 cells, lacking in Batf3−/− mice, were critical. Following intranasal immunizations isolated cDC1 cells from mLN exclusively promoted Rorgt^+^ T cells and IL-17, IL-21, and IL-22 production. Strong CD8 T cell responses through antigen cross presentation by cDC1 cells were also observed. Single-cell RNAseq analysis revealed upregulation of Th17-promoting gene signatures in sorted cDC1 cells. Gene expression in isolated cDC2 cells was largely unaffected. Our finding represents a major shift of paradigm as we have documented functional plasticity in cDC1 cells.

## Introduction

Migratory dendritic cells (DCs) play a key role as antigen-presenting cells (APC) for priming of CD4 and CD8 T cells in the draining lymph nodes^1^. These cells are CD11c^high^ and MHCII^high^ and migrate from the mucosal membrane to the draining lymph node after taking up antigen^2^. In this way antigen recognition following nasal immunization is mediated by migratory DCs that present processed peptides on MHC I or II molecules to naïve T cells in the T cell zone of the mediastinal lymph node (mLN)^3–5^. On the other hand, in steady state migratory DCs help maintain tolerance via induction of regulatory T cells in the draining lymph node^6^. Three main populations of migratory DCs have been identified and these belong to the classical DCs (cDCs), which are phenotypically distinct and have been linked to unique APC functions^7–9^. Differential surface expression of CD103 and CD11b provide the basis for lineage separation into CD103^+^ CD11b^−^, termed cDC1 cells, and CD103^+^ CD11b^+^ or CD103^−^ CD11b^+^, which are cDC2 double (cDC2DP) or single positive (cDC2SP) cells, respectively. The development of cDC1 and cDC2 cells is strictly controlled by gene expression as pre-DCs develop into these subsets in the bone marrow and they appear to be stable end-stages of differentiation with distinct transcriptional profiles^10,3,11–14^ The cDC1 cells, expressing *Irf8*, *Batf3*, *Id2,* and *Nfil3* genes, are known to cross-present MHC I-restricted peptides and, hence, stimulate CD8 T cells, but also CD4 T cell priming is effective as Th1 responses are induced^15–17^. The cDC2 cells, expressing *Irf4*, *Sirpα*, and *Zeb2* genes, on the other hand, can be divided into *Klf4*- or *Notch2*-dependent functional subsets and these prime CD4 T cells and promote Th2 and Tfh/Th17 differentiation, respectively^11,14,16,18,19^. Thus, immune responses against infection can be dependent on access to a specific cDC-subset in the tissues and, for example, protection against *Toxoplasma gondii* or *Schistosoma manosni* parasite infections require cDC1 and cDC2 cells, respectively^20,21^. Moreover, studies of protection against *C. rodentium* showed that cDC2 cells are required for IL-23 production whereas this function could not be replaced by cDC1 cells, supporting the notion that cDC1 cells are largely non-reduntant with cDC2 cells^22^. On the other hand tissue localization and environmental cues may define cDC-subset functions, because, for example, Th2 responses were stimulated by cDC2DP cells in the small intestine, while this was done by cDC2SP cells in the colon^12,21^. A recent study indicated that cDC2 subsets can change APC-function following adjuvant modulation^23^. It was demonstrated that cAMP-inducing agents, such as cholera toxin (CT) adjuvant, caused plasticity in *Klf4*-dependent cDC2 cells by repressing *Irf4*-expression, leading to a Th17- rather than Th2-promoting activity, a function normally associated with *Notch2*-expressing cDC2DP cells^16,23,24^. Moreover, cDC2 cells expressing *Tbet* have been reported to support Th1 cell development, which speaks in favor of functional plasticity in the cDC2 subset^12^. By contrast, functional plasticity was not observed with cDC1 cells following exposure to cAMP-inducing substances^23^. Thus, our current understanding of migratory cDCs is that they exhibit strict lineage commitment, but it appears that functional plasticity exist only among cDC2 cells, whereas it has not been described for cDC1 cells. So, for example, no study has demonstrated that cDC1 cells can induce Th17 responses, albeit Zelante et al reported that lung CD103^+^ DCs could prime Th17 cells, but no distinction between cDC1 and cDC2DP cells was made^25^.

To improve vaccine efficacy different strategies have been employed and efforts have focused on targeting vaccines to DCs^26^. The targeting has been achieved in many different ways, employing specific targeting elements, such as lectins or specific monoclonal antibodies (Mabs), or different antigen formulations, of which nanoparticles are a good example^27,28^. Whereas, the nanoparticles most often do not secure binding to a specific subset of DCs, conjugation of antigens to anti-CD103 or DEC205- Mabs can achieve selective targeting to cDC-subsets^5,29–31^. However, to be effective at priming of T cells most of the targeting strategies have required complementing toll like receptor (TLR)-ligand or anti-CD40 Mab stimulation^32^. Hence, targeting of vaccines to cDCs has not been found sufficient for improving vaccine efficacy without employing added adjuvant capacity to the formulation.

We have previously developed a fusion protein that accommodates the adjuvant activity of the enzymatically active CTA1-subunit of CT and a dimer of a D-targeting moiety, derived from *Staphylococcus aureus* proteinA^33^. The CTA1-DD adjuvant was developed to circumvent the toxic side effects of CT, including the accumulation in the brain that is associated with facial nerve paralysis following intranasal administration^34^. This fusion protein exerts comparable adjuvant effects to those found with intact CT^35^. In particular when given intranasally (i.n) CTA1-DD strongly augments cell-mediated as well as humoral immune responses^4^. Antigenic epitopes coupled or genetically fused to the CTA1-DD fusion protein or simply admixed with the adjuvant acquire strongly enhanced immunogenicity^36^. For example, the CTA1-3M2e-DD fusion protein, which carries the influenza M2e peptide, when given i.n, stimulates strong heterosubtypic protection against influenza virus infection^37^. The present study was undertaken to unravel how targeting of the adjuvant active CTA1-enzyme to CD103^+^ DCs could influence the immune response and which cDC subsets are responsible for the adjuvant effect. The choice of CD103-targeting was based on the fact that this membrane molecule allows for effective antigen uptake into the cDC and that it could potentially bind to both the cDC1 and cDC2DP subsets^29^. Therefore, we designed and expressed new fusion proteins with a single-chain antibody specific for CD103 (scFvCD103). The CTA1-scFvCD103 platform was then equipped with well defined peptides that were genetically fused into the molecule (CTA1-peptide-CD103) and used to analyze the peptide-specific T cell responses. This way we can now provide compelling evidence that CTA1 targeted to CD103^+^ cDCs changes the function of cDC1 cells to effectively prime for Th17 responses.

## Results

### The CTA1-CD103 fusion protein binds specifically to CD103+ dendritic cells

**Fig. 1:**
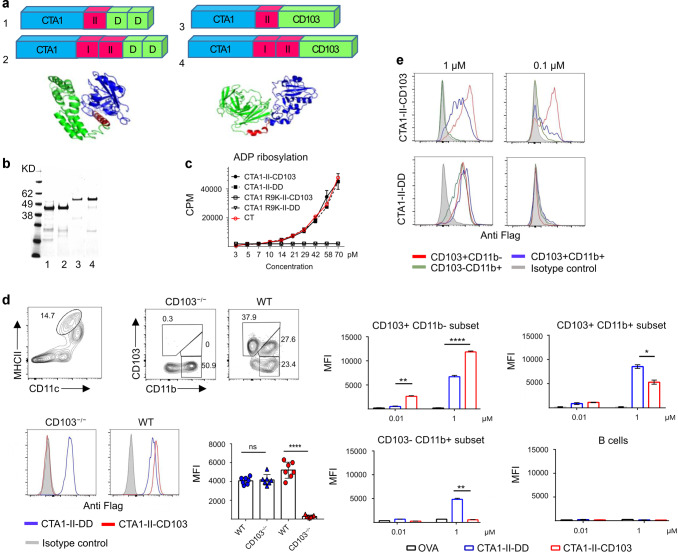
The scFv CD103 fusion protein specifically binds CD103^+^ DCs populations. Schematic representation and in silico model of the adjuvant active fusion proteins; CTA1-II-DD, CTA1-I/II-DD, CTA1-II-CD103, and CTA1-I/II-CD103 molecules, carrying the MHC I-restricted SIINFEKL-peptide and/or class II-restricted p323-peptide from ovalbumin (**a**). Western blot analysis of the CTA1-II-DD (1), CTA1-I/II-DD (2), CTA1-II-CD103 (3), and CTA1-I/II-CD103 (4) molecules (**b**). The ADP-ribosylating activity of CT holotoxin and the CTA1-enzyme or mutant CTA1R7K in the respective fusion proteins as assessed by the agmatine in vitro test. (**c**). Binding-specificity of the fusion proteins was assessed in vitro by gating on migratory DCs (MHC II^high^ CD11c^high^) from mesenteric LN (MLN) from WT or CD103−/− C57Bl/6 mice and flow cytometry with labeled DC-relevant Mabs (CD11c, MHC-II, CD103, CD11b) and labeled anti-Flag Mab to detect the fusion proteins used at 1 μM conc (**d**). Similar to D, but this in vitro analysis used WT migratory DCs or B cells (CD19^+^) from mediastinal LN (mLN) to assess the ability of the fusion proteins (MFI of labeled anti-Flag Mabs) at 1 and 0.1 μM concentrations to bind the different DC-subsets; CD103^+^CD11b^−^, CD103^+^CD11b^+^, CD103^−^CD11b^+^ or B cells using flow cytometry (**e**). Results are representative of three independent experiments with mean values±SD for means of triplicate cultures (**d**, **e**). Statistical significance was calculated using student *t* test with *p* value * *p* < 0.05, **p < 0.01, ***p < 0.001.

To achieve targeting of the CTA1-adjuvant to cDCs we fused the CTA1-encoding gene to the scFvCD103 gene and also incorporated gene segments encoding the MHC class I and/or class II-restricted peptides from ovalbumin; the SIINFEKL (I)- and/or p323 (II)-peptides, respectively (Fig. 1a, b)^35^. These constructs were then compared to our original CTA1-DD constructs, carrying the same inserted peptides, with regard to their ADP-ribosylating ability. We found that both the CTA1-II-CD103 and the CTA1-II-DD constructs exerted ADP-ribosylation, fully compatible with that of the CT-holotoxin (Fig. 1c). As expected, the CTA1R9K-mutation in both constructs obliterated the enzymatic activity completely (Fig. 1c)^33^. The binding-specificity of the fusion proteins to CD103^+^ DCs was assessed by using flow cytometry of migratory DCs (CD11c^high^MHC II^high^) isolated from mesenteric lymph nodes (MLN) from wild-type (WT) or CD103−/− mice. Both -DD and -CD103 targeted constructs bound migratory DCs, but the CTA1-II-CD103 fusion protein failed to bind DCs from CD103−/− mice (Fig. 1d, Supplementary Fig. 1). Of note, the CD103-constructs bound more effectively to CD103^+^ DCs than -DD constructs (Fig. 1d). Furthermore, freshly isolated DCs from the mLNs demonstrated binding of -CD103 constructs to both cDC1 (CD103^+^CD11b^−^) and cDC2 double-positive (cDC2DP)(CD103^+^CD11b^+^) subsets, while -DD constructs in addition bound cDC2 single positive (cDC2SP)(CD103^-^ CD11b^+^) cells (Fig. 1e). Again the CD103-targeted construct bound better to cDC1- than to cDC2DP cells, because of higher density of CD103 molecules on cDC1 cells, and more effectively than -DD fusion proteins (Fig. 1e). No binding to B cells in the mLN was observed, demonstrating that these CTA1-adjuvants, indeed, targeted migratory cDCs and preferentially cDC1 cells (Fig. 1e).

### CD8 T cell responses and cytotoxicity are enhanced by the CD103-targeted CTA1-adjuvant

**Fig. 2:**
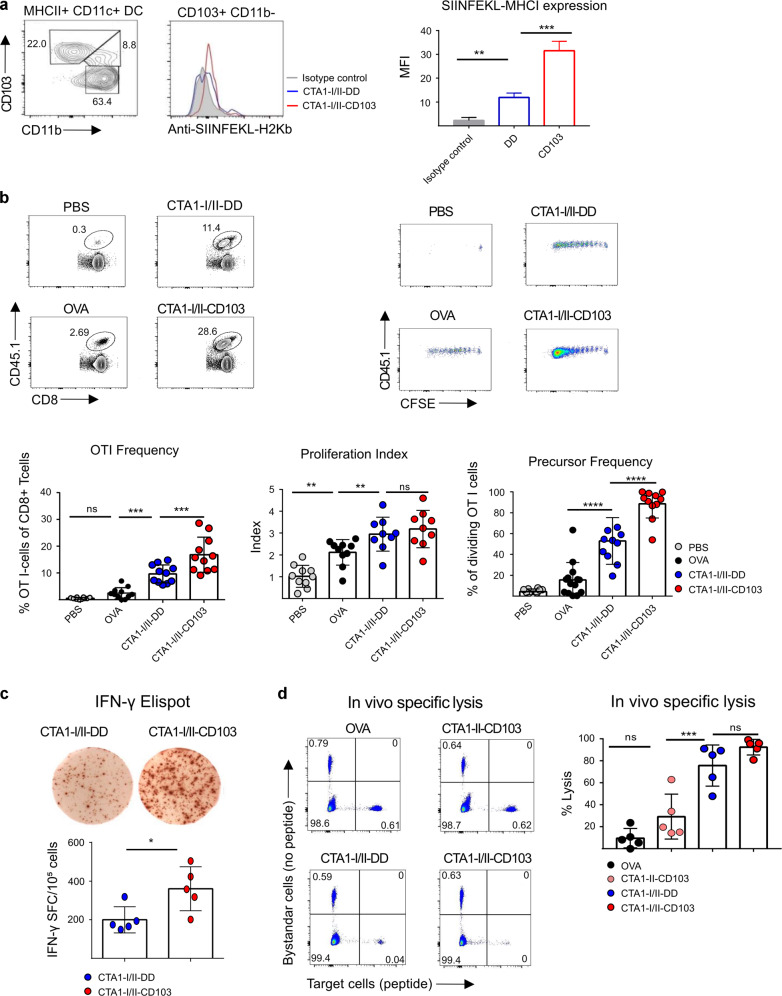
The CD103-targeted CTA1-adjuvant acts through cross-presenting cDC1 cells effectively priming CD8 T cells and CTLs. C57Bl/6 mice were i.n. administered with 5μM of OVA, CTA1-I/II-DD or CTA1-I/II-CD103, and 20 h later the frequency of migrating CD103^+^ CD11b^−^ cDC1 cells expressing SIINFEKL-peptide + MHC I complexes in the mLN was assessed using flow cytometry and a labeled complex-specific Mab and an isotype control Mab. MFI values are given as means±SD of three independent experiments (**a**). An adoptive transfer model with CFSE-labeled OT-I TCR Tg donor CD8 T cells (CD45.1^+^) injected into WT C57Bl/6 (CD45.2^+^) mice followed by a single i.n priming immunization with an equimolar dose (SIINFEKL peptide) of ovalbumin (OVA), CTA1-I/II-DD or CTA1-I/II-CD103. At 4 days post-immunization CD45.1^+^ OT I CD8 T cell proliferation was assessed in freshly isolated mLN cells by flow cytometry. The CFSE-dilution profiles were determined and values are given as the mean frequency ± SD of proliferating OT I cells of all CD8 T cells, proliferation index, and frequency of responding OT I CD8 T cells, as indicated (see M & M section) Representative dot-blots of three independent experiments giving similar results and values are given as means ± SD of 3–5 mice in each group (**b**). Determination of SIINFEKL-specific IFNy-ELISPOTs in OT I CD8 T cells stimulated with recall ovalbumin at 5 days following i.n immunizations with the fusion proteins. Values are given as means ± SD of 5 mice in each group and one representative experiment of three is shown (**c**). Induction of CTL responses in C57BL6 mice following i.n. immunization with a 5 μM dose of OVA, CTA1-I/II-DD, CTA1-I/II-DD, or CTA1- II-CD103 (control w/o SIINFEKL peptide) fusion protein. After 1 week mice were injected i.v. with CFSE-labeled splenocytes pulsed with 1 μM SIINFEKL peptide (Target cells) or un treated control splenocytes (Bystander cells). After 20 h freshly isolated mLN (mediastinal lymph node) cells were analyzed by flow cytometry for specific lysis of peptide expressing target cells . Values are means ± SD of two independent experiments giving similar results with 2–3 mice per group (**d**). Statistical significance was calculated using ANOVA with Dunnett’s post-test (**a**, **b**, **c**) or Student’s *t* test (**d**). ns; not significant; *p* values *p < 0.05, **p < 0.01, ***p < 0.001, ****p < 0.0001.

Because cDC1 cells (CD103^+^CD11b^−^) are known to cross-present and prime MHC class I-restricted CD8 T cell responses we investigated whether the CTA1-I/II-CD103 and CTA1-I/II-DD fusion proteins could stimulate SIINFEKL-specific CD8 T cell responses^7^. To this end C57Bl6 mice (Ly5.2^+^) were adoptively transferred with OT I T cells (Ly5.1^+^) that were labeled with carboxyfluorescein succinimidyl ester (CFSE) prior to i.v injections. Firstly we identified to what extent targeted migratory CD103^+^ CD11b^−^ DCs in the mLN carried SIINFEKL-peptide following i.n immunizations. We found that CD103-targeting of the CTA1-adjuvant induced a threefold higher surface expression of the SIINFEKL/MHC I complex in cDC1 cells compared to that provided by DD-fusion proteins, as assessed by flow cytometry and a labeled SIINFEKL/MHC I complex-specific Mab (Fig. 2a). Subsequently, we observed that priming of OT I cells was also more effective in the draining mLN following i.n immunizations with CD103- as compared to DD-constructs (Fig. 2b). We found that 90% of the OT I cells were primed, whereas an equimolar dose of the DD-construct was less effective, albeit much more effective than an equimolar dose of ovalbumin (Fig. 2b). The more effective priming of OT I cells resulted in higher frequencies of IFNγ-producing cells and enhanced cytotoxicity in vivo, as assessed by dividing the number of labeled target cells with control cells lacking the SIINFEKL-peptide (Fig. 2c, d). Intranasal immunizations with CTA1-II-CD103, without the SIINFEKL peptide, failed to stimulate cytotoxicity in OT I cells (Fig. 2d).

### CD103-targeted CTA1-adjuvant augments protection against influenza virus infection

**Fig. 3:**
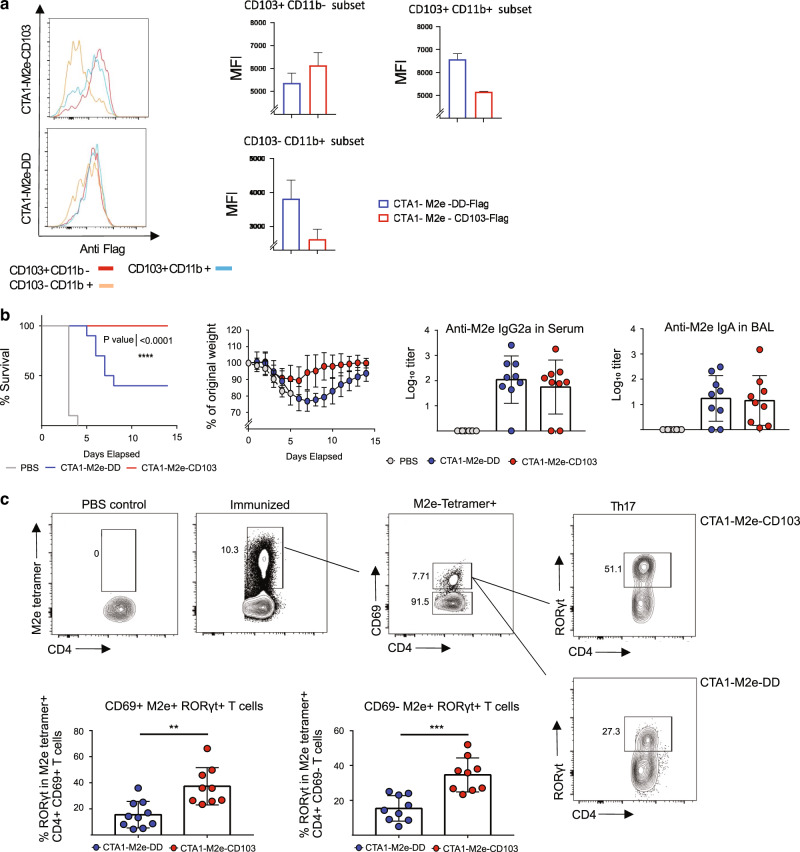
M2e-tetramer specific CD4 T cell protection against influenza virus infection following i.n immunizations with CTA1-M2e-CD103 fusion protein. Assessment of the binding ability of the M2e-specific fusion proteins -CD103 and -DD at 1 μM to bind to the three DC-subsets freshly isolated from mLN of Balb/c mice; CD103^+^CD11b^−^ (cDC1), CD103^+^CD11b^+^ (cDC2DP), CD103^-^CD11b^+^ (cDC2SP) in vitro using a labeled anti-Flag antibody to detect MFI of the bound fusion proteins to the different cDC subset populations as determined by flow cytometry (**a**). Balb/c mice were i.n immunized three times with 5μM of CTA1-M2e-CD103 (*n* = 10) or CTA1-M2e-DD (*n* = 10) and immune protection was determined 3 weeks after the final immunization by a live challenge infection with 4× LD_50_ of a highly virulent mouse-adapted PR8 strain^37^. The frequency of surviving animals and changes in body weight was monitored over time. Anti-M2e IgG2a (most protective antibodies) in serum and IgA in BAL after the challenge infection in one representative experiment of three giving similar results was assessed by ELISA and given as log_10_ titers ± SD (**b**). Representative FACS plots of M2e-tetramer-specific lung CD4 T cells isolated from i.n immunized or naïve control mice, as indicated. Prior to the challenge infection, the frequency of M2e-specific CD4 T cells that were CD69^+^ or CD69^-^ and the representation of Th17 cells (rorγt^+^) is given as % ± SD of all M2e-specific CD4 T cells (*n* = 3). Results are from three independent experiments giving similar results (**c**). Statistical significance was calculated using ANOVA with Dunnett’s T3 post-test analysis: ns; not significant, *p* values **p < 0.01, ***p < 0.001 and ****p < 0.0001.

Next we evaluated the adjuvant ability of the CTA1-enzyme when CD103- or DD-fusion proteins were used for i.n immunizations of Balb/c mice against influenza virus infections^38^. We have previously established that i.n immunizations with the CTA1-M2e-DD fusion protein effectively stimulate M2e-specific lung resident memory Th17 cells, which provided strong heterosubtypic protection against a live influenza virus challenge infection, while CTA1-DD adjuvant alone did not protect^37^. To this end we developed a CTA1-M2e-CD103 fusion protein to compare the protective properties of CD103- and DD-fusion proteins. In initial experiments using flow cytometry we tested the binding ability of M2e-constructs to isolated cDC-subsets from mLN and observed very similar binding properties to those we observed with the OVA-peptide carrying constructs, and, again, we identified a higher binding of the CD103-constructs to cDC1 cells (Figs. 3a, 1e). Following i.n immunizations in Balb/c mice we found that CD103-targeting was much superior to the DD-constructs in preventing a lethal influenza virus infection (Fig. 3b). Indeed, while 100% of the mice survived after i.n immunizations with CTA1-M2e-CD103 only 40% survived infection with the highly virulent A/PR/8/1934 (H1N1) strain (4 × LD_50_ dose) in the CTA1-M2e-DD immunized group (Fig. 3b). The stronger protection correlated with a higher frequency of M2e-tetramer binding Th17 cells (rorγt^+^) in the lungs in CD103- compared to DD- fusion protein immunized and challenged mice (Fig. 3c). As previously documented no M2e-specific CD8 T cells or Th1 cells were stimulated by the i.n immunization and a significant proportion of the M2e-specific Th17 cells were CD44^+^, CD11a^+^ and CD69^+^, indicative of lung resident memory cells, as we recently reported (Fig. 3c) ^38,39,40^. By contrast, no difference between specific serum anti-M2e IgG or local bronchioalveolar lavage (BAL) IgA antibody titers was detected in -CD103 and -DD- fusion protein immunized mice (Fig. 3b). Neither did testing of functionality in M2e-specific serum IgG antibodies, using an established cell-based ELISA as a replacement for assessing ADCC capacity, show a difference between -CD103 and -DD constructs (Supplementary Fig. 2A)^41,42^ Thus, it appeared that CD103-targeting was more effective than DD-constructs at promoting M2e-specific lung Th17-responses, which was associated with better protection against a live influenza virus infection, a mechanism partly dependent on IL-17 production, as demonstrated in our previous publications (Omokanye et al MI 2022) ^37^.

To extend the analysis we went on to compare the adjuvant efficacy of the -CD103-and -DD-based fusion proteins, using a fixed dose of soluble protein antigen, tetanus toxoid (TT) or NP-chicken gammaglobulin (NP-CGG) and decreasing doses of admixed adjuvant. In contrast to the M2e-specific responses these admixed protein immunogens together with CD103-targeted CTA1-adjuvant gave between 35 and 100-fold stronger antibody responses than -DD constructs (Supplementary Fig. 2B). Interestingly, even a dose as low as 0.014 μM of CD103-adjuvant was effective (Supplementary Fig. 2B). Therefore, we concluded that the CD103-targeting strategy for CTA1-adjuvant appeared significantly more efficacious at augmenting immunogenicity of admixed proteins compared to targeting with DD-constructs.

### Enhanced priming of Th17 cells in draining lymph nodes following i.n immunization with CD103-targeted fusion proteins

**Fig. 4:**
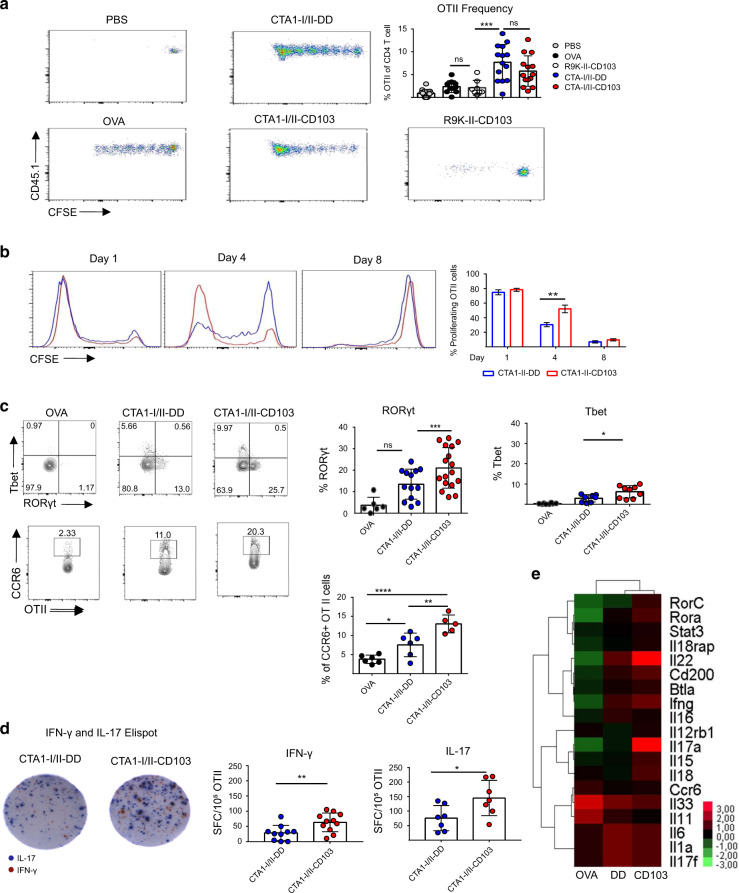
The CD103-targeted adjuvant effectively primes Th17 cells following i.n immunizations. Adoptive transfer i.v of CFSE-labeled OT II TCR Tg CD4 T cells (CD45.1^+^) to WT C57Bl/6 mice (CD45.2^+^) was followed by a single i.n immunization with 5 μM OVA, CTA1-II-DD or CTA1-II-CD103 the next day (equimolar doses of p323). On day 5 mice (*n* = 5) were analyzed for OT II cell proliferation in the mLN (mediastinal lymph node) by assessing the dilution of CFSE-label by flow cytometry. The enzymatically inactive CTA1R9K-II-CD103 fusion protein was evaluated for its adjuvant activity. The frequency of expanding OTII cells of all CD4 T cells is given as mean % ±SD of 3–5 mice in each group and pooled data from three independent experiments is shown. **a** The ability to prime OT II CD4 T cells in the draining mLN at different days post-immunization was assessed as in **a**. OTII cells were injected i.v. on days 1, 4, or 8 after i.n immunizations with the fusion proteins and values are given as means ±SD of 5 mice in each group (*n* = 2) (**b**). The differentiation into Th17 or Th1 OT II cells in the mLN following i.n immunization is given as the frequency of rorγt^+^, CCR6^+^ or Tbet^+^ of all OT II CD4 T cells. Values in each category are given as mean % ±SD of all OT II cells, as indicated, and these are representative of three independent experiments with 2–6 mice in each group (**c**). IL-17A and IFNγ-specific ELISPOT analysis of OT II CD4 T cell recall responses to p323 peptide was performed in triplicates by freshly isolated mLN cells following a single i.n immunization of the respective fusion protein.Values are given as mean±SD SFC/10^5^ cells of three independent experiments with 3-5 mice per group (**d**). Statistical significance was calculated using ANOVA with Dunnett’s posttest (**a**, **b**) or Student’s *t* test (**c**). *p* values are given as; *p < 0.05, **p < 0.01, ***p < 0.001, ****p < 0.0001. Finally, OT II cells from i.n immunized mice were subjected to a total RNAseq analysis using pooled samples from mLN from three mice. The heat map shows relative expression of Th17-relevant genes (*rorc, rora, IL-17a, IL-22, Ccr6*) in the different groups; OVA, CTA1-I/II-CD103 and CTA1-I/II-DD. This is a representative analysis of two independent experiments giving similar results (**e**).

To follow up with a more detailed investigation of Th17-priming after i.n immunizations we employed ovalbumin-specific (p323) OT II TCR transgenic CD4 T cells in an adoptive transfer model^43^. Similar to the M2e-specific CD4 T cell response, experiments with OT II cells confirmed that CD103-targeting of the CTA1-adjuvant effectively stimulated CD4 T cell proliferation in vivo (Fig. 4a). As expected, an enzymatically inactive mutant CTA1-molecule (CTA1R9K-II-CD103) was ineffective at stimulating OT II cell proliferation, in agreement with our previous studies (Fig. 4a)^37^. Noteworthy, the CD103-targeting itself did not exert adjuvant functions, attesting to that it was the enzymatic activity of CTA1 that was crucial for adjuvanticity. Interestingly, CD103-targeted CTA1-adjuvant was more effective at retaining a strong priming ability even at 4 days following immunization (Fig. 4b). Furthermore, the CD103-targeted fusion protein effectively promoted a Th17 cell response in OT II cells in the mLN, albeit Tbet^+^ Th1 cells were also induced (Fig. 4c). Also Th2 and Tfh cells were induced but less frequent than in OVA-only or DD-construct immunized mice (Supplementary Fig. 3A–C). Roughly 65% of the CD4 T cells were Th17 cells while Th1, Th2 and Tfh cells together comprised 35% (Supplementary Fig. 3D). ELISPOT-detection of IL-17 SFC or cytokine assessments in culture supernatants from isolated OT II cells from mLN of i.n immunized mice confirmed a strong IL-17A response, while IFNγ producing cells were 3-fold lower, but still augmented compared to an equimolar dose of ovalbumin (Fig. 4d, Supplementary Fig. 3C). A complementing gene expression analysis of isolated OT II cells from mLN of fusion protein i.n immunized mice attested to a prominent Th17 cell mRNA signature with upregulated expression of *rorc, rora, IL-17a*, and *IL-22* compared to that found with OT II cells from OVA-only immunized mice (Fig. 4e)^44^. Taken together, CD103-targeting of the CTA1-adjuvant favored Th17 cell differentiation in mLN following i.n immunizations.

### The CTA1-adjuvant is acting through cDC1 cells in CD103-targeted fusion proteins

**Fig. 5:**
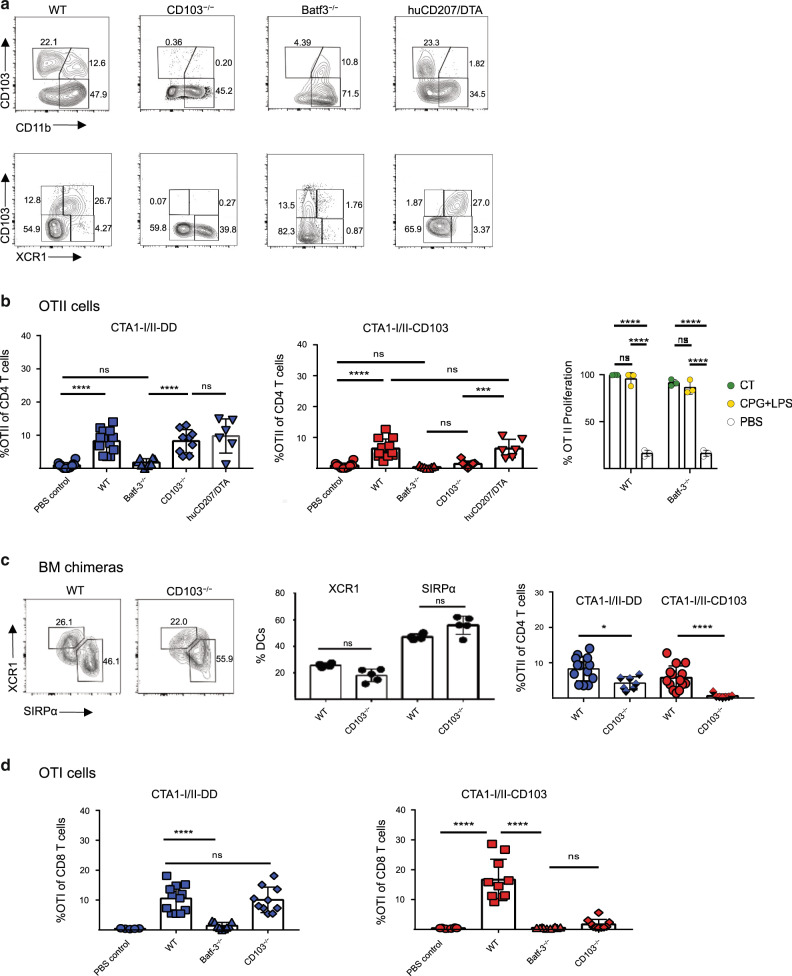
The CTA1-II-CD103 adjuvant effect is mediated via cDC1 cells. Mice with different restrictions in their DC-subset repertoire were immunized i.n following adoptive transfer of CFSE-labeled OT II cells (CD45.1^+^) according to the protocol used for Fig. 4. WT C57BL/6, transgenic CD103−/−, BATF3−/− or huCD207/DTA mice and littermate controls were immunized with 5 μM CTA1-II-DD (blue) or CTA1-II-CD103 (red) and migratory DCs (MHC^high^, CD11c^high^) and OT II cells were isolated and analyzed by flow cytometry after 20 h (cDCs) and 4 days (OT II cells), respectively. DC-subsets were identified using Mabs specific for CD103, CD11b and XCR1, as indicated. Representative dot-blots of DCs in mLN (mediastinal lymph node) following i.n immunizations are given for each mouse strain (**a**). OT II cell proliferation (dilution of CFSE labeling) was determined following a single i.n immunization and values are given as mean % ±SD of OT II cells of all isolated CD4 T cells in the respective group (*n* = 3–5) and representative of three independent experiments giving similar results . Control experiments in Batf3−/− mice for detection of cDC2-mediated OT II cell priming using OVA + CT- or LPS+CpG adjuvants given i.n (right panel). Values represent % OT II cells undergoing significant proliferation in response to adjuvanted or non-adjuvanted (PBS) responses to OVA (Histograms in Supplementary Fig. 4A (**b**). Bone marrow chimeric cDC1 (CD103−/−) and cDC2 (CD103^+/+^) mice were generated by reconstituting irradiated littermate C57Bl/6 mice with 50/50% BM from Batf3−/− and CD103-/- or WT mice (7.5 million BM cells /per mouse). The distribution of XCR1^+^ cells among cDC1 cells from reconstitutions with CD103−/− or WT BM (*n* = 4) is shown in the dot-blot. Following adoptive transfer of OT II cells (CD45.1^+^) into the chimeric mice and i.n immunizations as in **a** we show mean % ±SD of responding OT II cells of all isolated CD4 T cells in mLN of the respective group (*n* = 3–5) in two independent experiments (**c**). Adoptive transfer of OT I cells into mice with different cDC-deficiencies as in **a**, followed by i.n immunization with the fusion proteins and assessments of the efficiency of cross-presentation and priming of MHC class I restricted CD8 T cell responses. OT I cell (CD45.1^+^) responses were determined and values are given as mean % ±SD of all isolated CD8 T cells in the respective group (*n* = 3–5); pooled data from three independent experiments giving similar results (**d**). Statistical significance was calculated using ANOVA with Dunnett’s posttest and *p* values are given as; * *p* < 0.05, ***p < 0.001, ****p < 0.0001.

To address which subset of CD103^+^ DCs, cDC1 or cDC2DP cells, that the CD103-targeted CTA1-adjuvant was acting through we undertook experiments in genetically defined mice lacking distinct cDC subsets. To this end we employed Batf3−/− mice, lacking cDC1 cells, and huCD207/DTA mice, which have been found to be deficient in cDC2DP cells in the small intestinal lamina propria (LP) and MLN^45,46^. In addition, CD103-/- and WT C57Bl/6 mice were also included in the analysis. We confirmed that Batf3−/− mice lack cDC1 cells in the mLN and the huCD207/DTA mice did not have cDC2DP cells in the migratory cDC populations at this inductive site (Fig. 5a). Conversely, Batf3−/− mice had migratory cDC2DP cells and huCD207/DTA mice had intact cDC1 (XCR1^+^CD11b^−^) cells in the mLN (Fig. 5a). Moreover, CD103−/− mice had cDC1 (XCR1^+^CD103^-^) cells in the mLN. The cDC2SP (CD103^-^CD11b^+^) DC subset was present in all three mouse strains (Fig. 5a). We predicted that if the CD103-targeted CTA1-adjuvant was dependent on the cCD1 cells it would not work in Batf3-/- nor in CD103−/− mice for priming of OT II T cells. Following a single i.n immunization after adoptive transfer of OT II CD4 T cells we found that, indeed, CD103-targeted CTA1-adjuvant failed to stimulate OT II T cells in Batf3−/− as well as in CD103−/− mice (Fig. 5b). Whereas DD-constructs effectively primed OT II CD4 T cells in CD103−/− mice, they, unexpectedly, failed to do this in Batf3−/− mice, suggesting that also DD-constructs preferentially mediated their priming activity through cDC1 cells (Fig. 5b). Importantly, when CT or LPS + CpG adjuvants were used in Batf3−/− mice the proliferative response in OT II cells to i.n immunizations with OVA was significant, attesting to that cDC2-mediated CD4 T cell priming appeared intact in these mice (Fig. 5b and Supplementary Fig. 4a)^47^. Moreover, both CTA1-adjuvants effectively primed OT II CD4 T cells in the absence of cDC2DP cells in the huCD207/DTA mice (Fig. 5b). The dependence on cDC1 cells was further confirmed in bone marrow (BM) chimeric littermate WT mice (50% Batf3−/− and 50% CD103−/− or WT BM) that hosted both cDC1 and cDC2 cells, but lacked expression of CD103 on cDC1 cells (Fig. 5c). In these mice DD-constructs were effective, while -CD103-targeted CTA1-adjuvant failed to function (Fig. 5c). With regard to cross-presentation we found that, priming of OT I CD8 T cells was lost in Batf3−/− mice with both CD103- and DD-targeted SIINFEKL-carrying constructs (Fig. 5d). Taken together, these experiments clearly demonstrated that the targeted CTA1-adjuvants mediated their enhanced priming effects on CD4 T cells through cDC1 cells, while cDC2DP and cDC2SP cells appeared to be dispensible.

### Isolated cDC1 cells from intranasally immunized mice effectively induce Th17 cell differentiation ex vivo

**Fig. 6:**
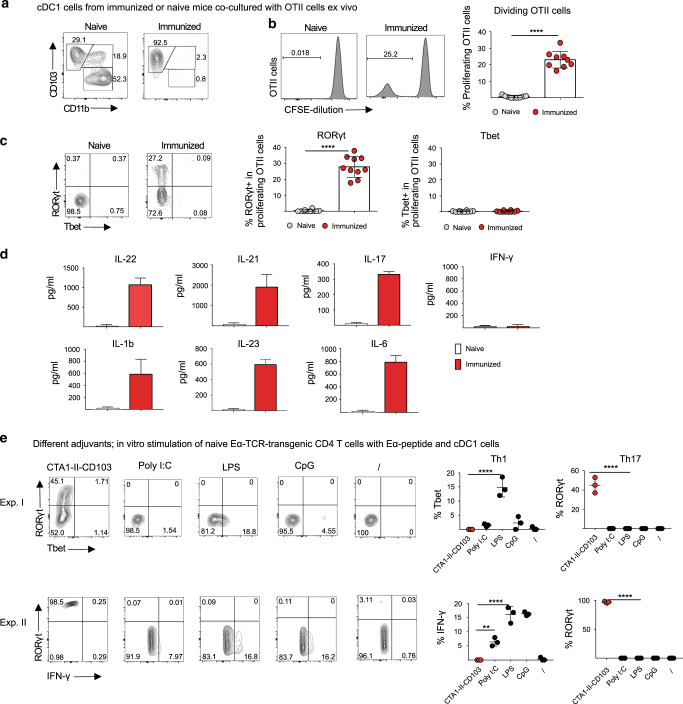
CTA1-exposed cDC1 cells are potent inducers of Th17 differentiation. Single cell suspensions of immunized or unimmunized mLN (mediastinal lymph node) mononuclear cells were highly enriched (>90%) for cDC1 (CD103^+^, CD11b^−^) cells by positive selection using biotinylated XCR1 Mabs at 20 h following an i.n dose of 5 μM CTA1-II-CD103. The enrichment strategy is shown with untreated cDCs from unimmunized mice and sorted cDC1 cells from mLN of immunized mice (**a**). Enriched migratory cDC1 cells (MHC^high^, CD11c^high^ XCR1^+^) from unimmunized or immunized mice were incubated in triplicates for 3 days with freshly isolated OT II T cells (1:10) labeled with CFSE and the histogram shows proliferating OT II cells in cultures with cDC1 from immunized or unimmunized mice and values are given as mean % ±SD of pooled cDC1 cells from 3–5 mice in each group and two independent experiments (**b**). The frequencies are given as mean % ±SD of Th17 (rorγt^+^) and Th1 (Tbet^+^) CD4 T cells on day 3 in these cultures of proliferating OT II cells with unimmunized as opposed to immunized cDC1 cells (**c**). Cytokine production, as indicated, in these cultures with unimmunized or immunized cDC1 cells is given in pg/ml ±SD for each group (**d**). In vitro triplicates cultures with enriched cDC1 cells from unimmunized mice were co-cultured with naïve Eα-peptide-specific TCR Tg CD4 T cells (1:10) and 1 μM Eα-peptide together with or without different adjuvants added simultaneously; CTA1-II-CD103 (1 μM), Poly I:C (10 μg/ml), LPS (10 μg/ml), CpG (2.5 μg/ml) or unstimulated (\), as indicated. The frequency of Rorγt^+^ or Tbet^+^ Eα-specific TCR Tg CD4 T cells in cultures was determined on day 3 and the frequency of intracellular IFNγ^+^ cells was assessed by flow cytometry using labeled specific antibodies. Two independent experiments (I and II) are shown giving similar results (**e**). Statistical significance was calculated using ANOVA with Dunnett’s posttest and *p* values are given as; ****p < 0.0001.

Because of the outcome of these in vivo experiments we turned to more rigorously controlled in vitro experiments. Given that our results suggested that cDC1 cells were mediating the effect of CD103-targeted CTA1-adjuvant we asked if highly enriched migratory cDC1 cells from the mLN following i.n immunization could drive OT II CD4 T cell proliferation and Th17 differentiation ex vivo (Fig. 6a). Indeed, only cDC1 cells from immunized mice stimulated cell division (CFSE-dilution) and Th17 (rorγt^+^) cell differentiation, whereas cDC1 cells from unimmunized mice failed to do so (Fig. 6b). Thus, only cDC1 cells exposed to the fusion protein in vivo could activate the OT II cells, strongly supporting the observations in WT and Batf3−/− mice that cDC1 cells are the critical mediators of Th17 responses in our model system. Importantly, we observed no Tbet^+^ OT II CD4 T cells in the cultures and no IFNγ was produced (Fig. 6c). Rather, enriched cDC1 cells from i.n CTA1-II-CD103 immunized mice stimulated significant levels of IL-17, IL-21 and IL-22, indicative of Th17 cells, as well as IL-1β, IL-6 and IL-23, cytokines that drive Th17 development (Fig. 6d). In vitro cultures enriched for cDC1 cells from mLN from unimmunized mice developed rorγt^+^ OT II CD4 T cells only when CTA1-II-CD103 fusion protein was added to the cultures (Supplementary Fig. 4B). In addition, enriched cDC1 cells from mLN together with Eα-specific TCR Tg CD4 T cells cultured with Eα-DD antigen and CTA1-II-CD103 or other adjuvants; Poly I:C, LPS and CpG, revealed that only the CTA1-adjuvant induced rorγt^+^ Th17 cells in vitro (Fig. 6e)^5^. By contrast, the TLR-dependent adjuvants stimulated IFNγ production, indicative of Th1 (Tbet^+^) responses, as predicted (Fig. 6e)^48^. CD4 T cells without co-culture with cDC1 cells failed to respond to the different stimuli (Supplementary Fig. 4B). Thus, highly enriched cDC1 cells responded to the ADP-ribosylating CTA1 adjuvant with Th17 priming, while TLR-dependent adjuvants promoted Th1 and IFNγ-responses in vitro.

### Single cell RNAseq analysis reveals strong transcriptional impact on cDC1 cells of CTA1-adjuvant

**Fig. 7:**
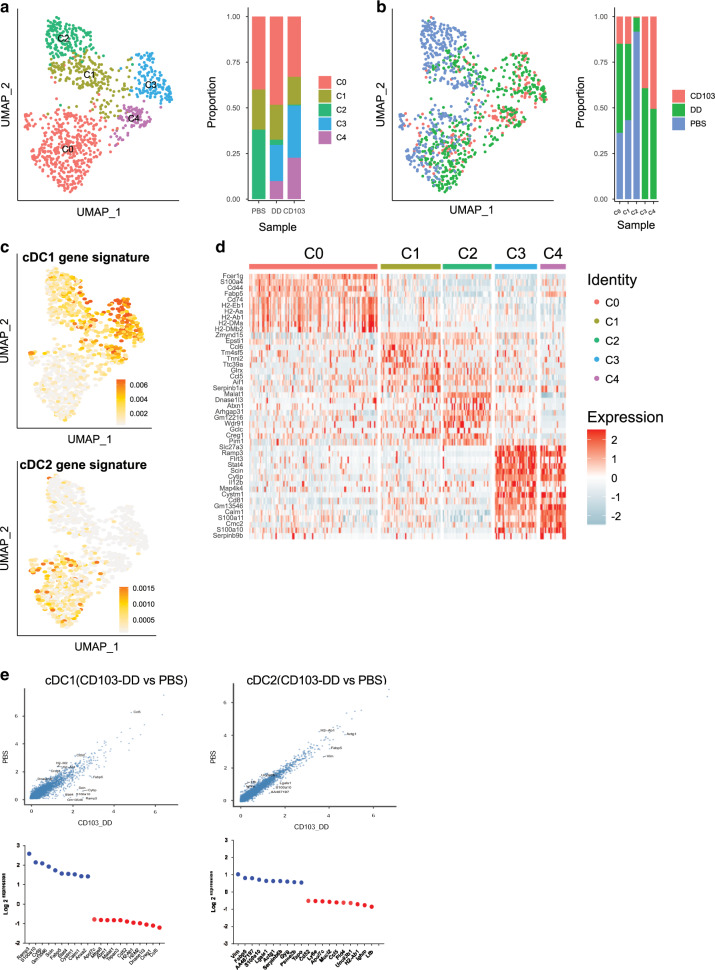
Single-cell RNAseq analysis of migratory DCs in mLN following i.n immunizations with a CD103-targeted adjuvant. Migratory cDC-subsets that had been exposed to a single dose i.n of 50 μM CD103 or -DD constructs with incorporated Eα-peptide were identified by labeled Yea-Mab that binds Eα−peptide + MHC class II (I-A^b^)^39^. This strategy secured that only cDCs exposed to CTA1-adjuvant in vivo were included in the analysis and these were compared to cDCs from unimmunized mice. Migratory cDCs were sorted by FACS and subjected to a scRNAseq analysis using the 10× Chromium platform. Cluster analysis was performed using Seurat and the UMAP-representation of the cDC-subsets from naïve PBS, CTA1-II-DD or CTA1-II-CD103 treated mice is depicted and the cluster or treatment distributions of individual cDCs is given (**a**, **b**). Distribution of cDC1 and cDC2 cells among the analyzed cDCs, according to the UMAP, based on cDC1 and cDC2 subset-restricted gene definitions (**c**). Heat map showing the 25 top DEG in the different clusters of migratory cDCs following immunization (**d**). Top 20 differentially expressed genes (DEG) in CTA1-exposed (DD- + CD103 constructs) as opposed to unimmunized cDC1 (left panels) and cDC2 subsets (right panels) (**e**).

We investigated to what extent migratory DCs changed gene expression profiles as a consequence of CTA1-adjuvant targeting using the 10X Chromium platform and single cell RNAseq (scRNAseq) analysis^49^. To this end C57Bl/6 mice received a single i.n dose of CTA1-II-CD103 or CTA1-II-DD and FACS-sorted migratory DCs in the mLN were subjected to scRNAseq analysis. The gene expression profiles were compared with those of migratory DCs from unimmunized PBS-treated control mice. To secure that we analyzed DCs that had bound the fusion protein we used fusion proteins that carried an Eα-peptide, so that we could identify the targeted cells as they expressed Eα-peptide/MHC II complexes using labeled Yea-Mab (anti-Eα peptide /MHC II complex) and flow cytometry^5^. This way we exclusively analyzed migratory DCs targeted by CTA1-adjuvant in the mLN and compared their gene expression profiles with those obtained from cDCs from unimmunized PBS-treated control mice. Of note, the isolated control cDCs from unimmunized mice represent naturally activated migratory cells^12^. After 24 h following i.n administration of 50 μM doses of CTA1-Eα-CD103 or-DD we isolated and FACS-sorted Yea-Mab^+^ DCs into high purity. After quality control and filtering we used the Seurat R toolkit to perform unsupervised clustering of the DCs based on differentially expressed genes (DEG) and the data was visualized using uniform manifold approximation and projection (UMAP) in two dimensions (Fig. 7a, b)^50^. We analyzed a minimum of 500 cells in each category. The migratory DCs were distributed in five clusters and a distinct separation of cDC1 and cDC2 cells into these clusters was evident using published gene lists defining the subsets (Fig. 7c, Supplementary Fig. 5A, B)^12^. A striking observation was that migratory cDC1 cells underwent a more dramatic change following binding with CTA1-adjuvant than the corresponding cDC2 cells (Fig. 7a–c). Thus, both CD103- and DD-targeted cDC1 cells were found in clusters 3 and 4, while cDC1 cells from unimmunized mice were restricted to cluster 1 and 2 (Fig. 7a, b). By contrast, cDC2 cells were found in cluster 0 and both CTA1-exposed and control migratory DCs inter-mingled in this cluster (Fig. 7b, c). Hence, the most dramatic shift occured in cDC1 cells, while cDC2 cells appeared less affected by the CTA1-adjuvant. The heat-map of the top 25 differentially regulated genes in migratory DCs from control or CTA1-treated mice also identified that cDC1 cells were the most affected, as evident in clusters 3 and 4 as opposed to gene expression in clusters 1 and 2 (Fig. 7d). Even though -CD103 constructs were found to be more effective than DD-constructs at targeting cDC1 cells, the difference in gene expression profiles was minimal between the two with only *Rgs-1*, *Cd83, Rel* and *Ccr7* genes differentially expressed (Supplementary Fig. 5C). Nevertheless, both targeting constructs were effective at promoting CTA1-mediated immunomodulation of cDC1 cells, while cDC2 cells were less affected (Fig. 7e). A set of the 10 most significantly DEG were identified in cDC1 cells and these genes were linked to activation and CD4 T cell priming functions (Fig. 7e). Of note, *Stat4*, *Scin* and *Cytip* genes have all been associated with DC activation/migration and *Fabp5* gene expression has linked fatty acid metabolism to Th17 differentiation ^51,52^.

**Fig. 8:**
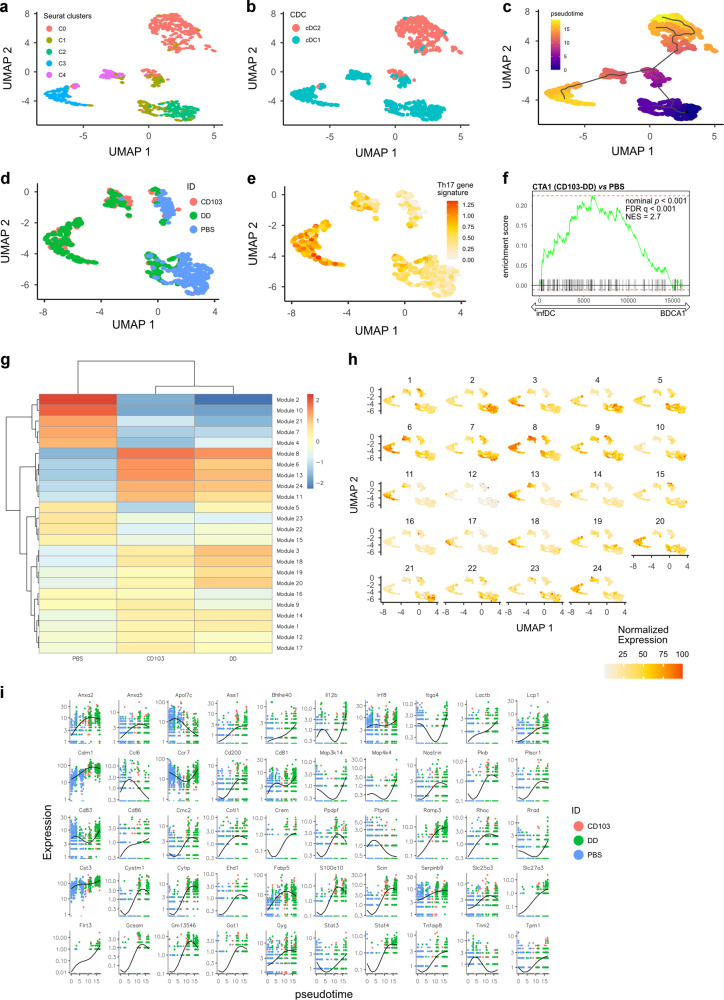
Pseudotime trajectory of differentially regulated genes in CTA1-exposed CD103-targeted cDCs reveals plasticity of cDC1 cells and ability to induce Th17 cells. The continued scRNAseq analysis explored the data set using Monocle 3 with which we established a developmental trajectory of the CTA1-exposed cDC1 cells. The data set from Seurat was analyzed in Monocle 3 and depicted in the UMAP plot (**a**) and as represented after using the gene signatures of cDC1 and cDC2 cells, as in Fig. 7e (**b**). We undertook a pseudotime trajectory analysis of the data with starting node in cluster 0 (**c**). The distribution of cDC1 cells in the UMAP following different i.n immunizations was depicted (**d**). The expression level of a Th17-promoting gene signature (*IL-1b, IL-6, Tgfb1, Tgfb2, Tnfsf4(*Ox40L*), Tnfrsf9(*CD137*), IL-12b(*p40*), Timd4, Cd80, Cd86*) was employed to follow gene expression from naïve PBS to CTA1-exposed cDC1 cells along the trajectory (**e**). An extended gene set enrichment analysis (GSEA) using a gene list taken from a publication of human inflammatory DCs, reported to promote Th17 responses, was compared with the list of co-differentially expressed genes found in CTA1-exposed cDC1 cells^55^ (**f**). The distribution of gene modules which significantly vary across pseudotime is depicted (**g**–**h**). Selected genes from the modules in **g** are represented in the trajectory of cDC1 cells (**i**).

We generated a pseudotime trajectory of gene expression in cDC1 cells using the Monocle 3 program (Fig. 8a–c)^53^. This analysis indicated that cDC1 cells had developed a gene expression profile that could represent a transition of function from the unmanipulated cDC1 cells to those exposed to the CTA1-adjuvant (cluster 3 and 4). (Fig. 8a–d). Strikingly, when subsetting on cDC1 cells and applying a selection of critical genes known to be driving Th17 cell differentiation we observed a clear increase in such genes along the pseudotime trajectory (Fig. 8e)^54,55^. Indeed, genes encoding co-stimulatory and Th17-promoting factors (*IL-1b, IL-6, Tgfb1, Tgfb2, Tnfsf4 (*Ox40L*), Tnfrsf9 (*CD137*) IL-12b (*p40*), Timd4, Cd80, Cd86*) were distinctly up-regulated in adjuvant exposed, as opposed to unimmunized control, cDC1 cells (Fig. 8d, e, Supplementary Fig. 5D). An extended gene set enrichment analysis (GSEA) using a gene list taken from a publication of human inflammatory DCs, reported to promote Th17 responses, was performed with the list of co-differentially upregulated genes found in CTA1-exposed cCD1 cells^55^. The GSEA showed significant enrichment of DEG that previously have been linked to Th17 promoting activity (Fig. 8f). To further the analysis, the subsetted cDC1 population was analyzed for genes that change across the pseudotime. Genes were then grouped into modules using Louvain community analysis. Several activation and signaling pathways were involved as the cDC1 transitioned from the PBS cluster state to the most active CTA1-exposed cells (cluster 3) and this was seen with the modules showing most dramatic changes in gene expression (Fig. 8g–h, Supplementary Excel files). For example, modules hosting *Stat3, Rel, Irf8, Crem* and *Stat4* and genes encoding activation, cell adhesion and co-stimulatory molecules (*Anxa5, Bhlhe40, Cd86, Cd200, Cytip, Itga4, Flrt3, Scin, Tnfaip8, ox-40l*) or genes involved in cell division, motility and survival (*Cd81*, *Cd83, Ccr7, Map4k4*) and cytokine and chemokine relevant genes (*Anxa2*, *Il-12b, Map3k14*) were significantly up-regulated in CTA1-adjuvant exposed cDC1 cells (cluster 3 and 4), consistent with a strong CD4 T cell and Th17 priming function (Fig. 8i). Furthermore, genes linked to metabolism were upregulated in these modules; *Slc27a3*, *Fabp5* gene, fatty acid metabolism, and *Ass1*, L-arginine and urea metabolism, *Calm1* ans *S100a10*, involved in calcium metabolism (Fig. 8i)^51,52,56–59^. Antigen-cross presentation has been linked to a high expression of *Serpinb9*, which was clearly up-regulated in cDC1 cells in CTA1-exposed cluster cells (Fig. 8i)^60^. Thus, exposure to CTA1-adjuvant dramatically changed the gene expression pattern in cDC1 cells leading to stimulatory functions that promoted Th17-differentiation in primed CD4 T cells as well as an improved ability to cross-present and stimulate CD8 T cell responses.

## Discussion

Previously published studies have indicated that the two lineage-specific cDC-subsets are to be viewed as functionally specialized and largely non-reduntant because the cDC1 subset promotes Th1 cell development and cross-presentation to CD8 T cells, while cDC2 cells are responsible for Th2 and Th17/Tfh cell priming^1,10–12,16,17,21,61,62^. However, the present study challenges this notion by demonstrating that cDC1 cells can, under certain conditions, prime Th17 cells as we show following i.n immunizations with the CTA1-peptide-CD103 fusion protein. We provide unequivocal evidence demonstrating that the CTA1-peptide-CD103 fusion protein, indeed, acts through cDC1 cells and not through cDC2DP cells, albeit the latter cells also express CD103. Several lines of evidence support this interpretation of our results; 1) Batf3-deficient mice, which lack cDC1 cells, fail to respond to the fusion protein, 2) in vitro cultures of naïve CD4 T cells with highly enriched cDC1 cells from i.n immunized mice respond with Th17 development and IL-17, IL-21 and IL-22 production, 3) highly enriched cDC1 cells produce IL-1β, Il-6 and IL-23 upon stimulation with the fusion protein in vitro, and finally 4) a scRNAseq analysis of freshly sorted cDC1 and cDC2 cells revealed that cDC1 cells, rather than cDC2 cells, undergo extensive CTA1-enzyme dependent modifications of gene expression, some which have been linked to a Th17 priming function in earlier studies^23,51,55,63,64^. This ability of CTA1-adjuvant appears to be quite unique as CpG, poly I:C and LPS, exerting their actions via Myd88/TRIF-signaling pathways and different Toll-like receptors (TLRs), promote Th1 differentiation, in agreement with previous reports^48,65^. The CTA1-adjuvant effect on cDC1 cells was dependent on its ADP-ribosylating ability since an enzymatically inactive mutant did not exert adjuvant function, which we have also reported earlier^35^. Thus, the present study has unraveled that functional plasticity can be exhibited by cDC1 cells, which were found to prime for Th17 cell differentiation. Using an ADP-ribosylating CD103-targeted CTA1-adjuvant we achieved M2e-specific Th17 differentiation and strong protection against influenza virus infection.

In earlier work we targeted antigen to CD103^+^ APCs via conjugation to anti-CD103 Mab and found that both CD4 and CD8 T cells could be effectively primed^29^. Exploiting the CD103-targeting element for delivery of a strong mucosal adjuvant, such as CTA1, was, therefore, a natural next step in this strategy. Apart from antigen-delivery we achieved a strong immunoenhancement of the APC function in CD103^+^ cDC cells, resulting in augmented priming ability of both CD4 and CD8 T cells, without adding a separate adjuvant because the fusion protein itself harnessed the CTA1-adjuvant^35^. Hence, the fusion protein provided targeting, immunoenhancement and delivery of vaccine epitopes, in one molecule. In agreement with our earlier work with anti-CD103 Mab targeting, the ScFv-approach exhibited strict dependence on CD103-expressing cDCs for its immune stimulating effects^29^. This was evident from the lack of an effect in CD103−/− mice, and, perhaps, best noted in chimeric mice that hosted cDC1 cells, which were CD103-deficient, and failed to respond. Thus, the target population was restricted to cDC1 and did not depend on cDC2DP cells, despite that the latter carry CD103. In fact, huCD207DTU mice that were deficient in cCD2DP cells in mLN demonstrated full adjuvant and antigenic properties in response to the fusion protein, while Batf3−/−mice, lacking cDC1 cells, did not respond to CD103- or DD-targeting, but reacted to other adjuvants given intranasally ^45–47^.

To secure that we investigated migratory cDCs that had bound and taken up the fusion protein following i.n administration we employed FACS-sorting using labeled Yae^+^ Mab that detects the Eα-peptide on the cDC cell surface^5^. This way we could identify the exact subset of migratory cDCs that were affected by CTA1-adjuvant and which were instrumental for priming of the CD4 T cells in the draining mLN. Noteworthy, for comparison we sorted migratory cDCs from mLN from unimmunized mice, cells that had been naturally activated and matured, prior to trafficking to the mLN. The scRNAseq analysis was highly revealing and identified a substantial modification of genes expressed in immunized as opposed to unimmunized cDC1 cells, while cDC2 cells were less affected and found within the same cluster in the UMAP. The cDC1 cells from unimmunized mice distributed mainly to clusters 1 and 2, while a majority of CD103-targeted cells were found in clusters 3 and 4. The greater impact on cDC1 cells agreed well with the absolute requirement for cDC1 cells, lost in Batf3-/-, for the adjuvant effect^45^. Thus, cDC1 cells were distributed in 4 different clusters and a pseudotime trajectory analysis identified significant gene expression changes in CTA1-exposed versus adjuvant-unexposed cDC1 cells. These changes affected genes associated with cell migration, cell signaling and co-stimulation. More specifically, we observed increased expression of genes associated with Th17-priming (*IL-1b, IL-6, Tgfb1, Tgfb2, Tnfsf4 (*Ox40L*), Tnfrsf9 (*CD137*) IL-12b (*p40*), Timd4, Cd80, Cd86*) along the trajectory in cDC1 cells exposed to CTA1-adjuvant^7,55^. Strikingly, a GSEA analysis based on gene lists from a previous publication of human inflammatory DC and a Th17-inducing function, supported our interpretation that the cDC1 cells had acquired a Th17 promoting gene signature following CTA1-exposure^55^. Noteworthy, the gene list used represented inflammatory DC that were thought to be derived from monocytes, genetically more like murine CD11b^+^ cDC2 cells and not cDC1 cells^14,55^. But, despite this our comparative analysis showed significant gene enrichment for a Th17-inducing function, similar to the “pro-Th17 gene signature” reported earlier for inflammatory DCs in humans, i.e CD163^+^DC3s,which are related to cDC2s^14,55^. Furthermore, the up-regulated expression of *IL-1b, IL-6, Tgfb1 and IL-12b (*p40*)* genes we found are clearly linked to a Th17 promoting function by cDCs^14,55^. Apart from the up-regulated expression of these known Th17-promoting genes we identified several DEG typifying activated cDCs^1,54,55^. For example, up-regulated expression of *Stat3*, *Stat4*, *Cytip*, *Scin*, *Ccr7, Anxa2, Cd86* and *Cd81* genes are all hallmarks of highly activated DCs and could greatly contribute to an enhanced T cell priming efficacy by facilitating cDC1 cell migration and/or antigen presentation in the immune synapse^56,58,66–69^. *Serpinb9* expression is a marker for antigen-cross presentation by cDC1 cells^60^. The interaction between tetraspanin CD81 and MHCII molecules is well documented in APCs, although CD81−/− mice have not been investigated for poor Th17 development, only impaired Th2-cell immunity and antibody production, have been reported^56,66^. Moreover, among the DEGs with strongest association to Th17-development, we find *Fabp5*, *Rel* and *Ox40L*^52,70^. Expression of *Rel* links cDC1 cells to the transcription factor NF-*k*B and signaling events that control *IL-23 p19* gene expression and in this way influences Th17 differentiation in CD4 T cells^70^. The involvement of fatty acid metabolism in Th17 differentiation has been documented before and appears to link DC activity to Stat3-signaling and Th17-differentiation in CD4 T cells^51,52^. *Tnfsf4*-expression, encoding OX40L, has been associated with autoimmunity and Th17 cell differentiation, but reported to be expressed only in cDC2 cells and not in cDC1 cells ^71,72^.

We have previously demonstrated that the lung resident M2e-specific Th17 memory cells convey strong protection against influenza virus infection following i.n immunization with CTA1-M2e-DD^37^. In the present study we improved immunogenicity of the fusion protein by changing the targeting element from DD to scFvCD103 and achieved significantly stronger protection compared to the DD-constructs. This way the efficiency in targeting the fusion protein to cDC1 cells was much improved, although we learned that both the -DD and -CD103 constructs relied on the cDC1 subset for their adjuvant functions. This latter observation was unexpected as cDC2DP cells were also found to bind the fusion proteins, but these cells failed to replace cDC1 cells in Batf3−/− mice. In particular, the -DD constructs, which bound cDC1 and cDC2 cells equally well, were found poorly effective in Batf3−/− mice. Similar to intact CT-adjuvant, both CTA1-fusion proteins were effective at augmenting CD8 T cell priming by cross-presenting cDC1 cells^73^. This ability has recently been successfully explored by us in a therapeutic vaccine model against tumor metastasis where we observed augmented tumor-specific CTL-induction, which agrees well with previous studies on CD103-targeted vaccine approaches and CT-adjuvant (M. Arabpour et al, unpublished data) ^74,75^.

Functional plasticity in cDCs has previously been observed within the cDC2 lineage subsets and was reported to depend on the level of expression of the *Irf4* and *Klf4* genes^23^. While increases in intracellular cAMP repressed these genes and modified the function of cDC2 cells to prime Th17 rather than Th2 responses, there were no modifying effects of cAMP-increases reported in splenic cDC1 cells^23^7. In the present study we found that CT given as an i.n adjuvant was effective in Batf3−/− mice attesting to a cDC2-mediated mechansims of action. By contrast, no dependency on cDC2 cells for the adjuvant effect of the targeted CTA1-adjuvant was observed and Lee et al had no effect of CT or other cAMP-enhancers in cDC1 cells^23^. Therefore, we may speculate that although CTA1 is the cAMP-inducing element in the CT holotoxin it is probable that the CTA1-fusion protein mediates adjuvanticity in the absence of cAMP-increases. Indeed, preliminary studies have indicated that the effect of the fusion protein may be independent of Gsα-expression and cAMP-induction in CD11c^+^ cells^35^. Additional experiments are required, though, to prove this point. Importantly, the CD103-targeting ScFv-element in itself had no adjuvant effect and could not promote Th17 cell development in vitro. This effect was rather a consequence of CTA1-actions on the cDC1 cells, which included enhanced co-stimulation and cytokine production with increased IL-1β, IL-6 and IL-23 levels in cultured cDC1 cells after exposure to the adjuvant. This was in contrast to the TLR-binding adjuvants, CpG, poly I:C and LPS, which all stimulated Th1 and IFNγ-production and they had no promoting effect on rorγt^+^ Th17- or IL-17 responses in cultured cDC1 cells^48,65^. To our knowledge the present study represents a major shift of paradigm as it demonstrates that cDC1 cells show plasticity and under certain conditions, can be inducers of Th17 responses, a function previously thought to be restricted to cDC2 cells. A previous study by Zelante et al documented Th17-inducing ability of lung CD103^+^ DCs in the context of fungal exposure, but these authors did not discriminate between cDC1 and cDC2 cells and their model for IL-2 producing cDCs, the D1 cell line, expresses cDC11b, rendering this cell the status of a cDC2DP cell^25^. The present study not only challenges the idea of a functionally non-redundent lineage-restricted cDC1-subset, but it is likely to have fundamental impact on future vaccine adjuvant development and Th17-mediated immune protection, in particular.

## Materials and methods

### Mouse strains and immunizations

Female mice, 6–10 weeks of age, were used for all experiments. C57Bl/6 (CD45.2^+^) and Balb/c wild-type (WT) mice were purchased from Janvier Labs (Paris, France). OT-II (CD45.1^+^), OT-I (CD45.1^+^), Eα-TCR Tg mice^76^ and Batf 3−/− mice were bred at EBM animal facility at the University of Gothenburg. HuCD207/DTA^46^ and *Itgea* (CD103−/−) knock out mice on the C57Bl/ 6 background (were bred at Lund University (Lund, Sweden) and transported to Gothenburg for experiments. Animal experiments were approved by the local Ethics Committee.

C57Bl/6 or Balb/c WT mice were immunized i.n with a single dose or three doses with 10 days apart, as indicated. Balb/c mice were used only for influenza virus protection studies because the M2e-peptide is restricted to H-2^d^^37^. For comparative studies of adjuvant efficacy between CD103- and DD-constructs immunizations were also performed i.p, as indicated, with admixed soluble antigens; 5 μg 4-Hydroxy-3-nitrophenylacetyl-Chicken Gamma Globulin (NP-CGG or tetanus toxoid (Statens Serum Institute, Copenhagen, Denmark).As alternative adjuvants in Batf3−/− mice we compared the effects of the fusion proteins with ovalbumin (OVA) given together with CT at 1 μg/dose or LPS + CpG at 10 μg doses. Fusion proteins and ovalbumin were given in 5 μM doses, unless stated otherwise. Serum and bronchoalveolar lavage (BAL) was sampled 6–8 days after the last immunization or 18 days after inoculation of virus and stored frozen at −20C until analyzed for specific antibody titers by ELISA.

### Influenza virus challenge infection

Immunized or PBS control Balb/c mice were subjected to a virus challenge infection. Briefly, 2–3 weeks after immunizations groups of ten mice were inoculated with an i.n dose of PR8 A/Puerto Rico/8/34 (H1N1) virus at 4 × LD50, (corresponding to 2.5 × 10^3^ TCID_50_). Morbidity (body weight) and mortality were monitored daily for 2 weeks^37^. Mice were sacrificed when reaching a weight loss >30%.

### Fusion proteins

CTA1-peptide-CD 103 fusion proteins were produced; Briefly, anti-CD103 single chain antibody (CD103 scFv) cloning and construction was done using regions from both variable heavy (VH) and light (VL) immunoglobulin chains from the anti-CD103 antibody produced by the M290 hybridoma. The desired regions from M290 VH and VL chains were RT-PCR amplified and sequenced. Amplified regions were linked together via the genetic sequence corresponding to the 4 GGGGS linker region to form scFv CD103. This was followed by linking a gene sequence corresponding to the cholera toxin A1 enzyme (CTA1) subunit or its mutated and enzymatically inactive CTA1(R9K) gene sequence which was fused N-terminally of the anti-CD103 scFv fragment. Constructs with peptides incorporated into the fusion proteins were designed as follows; fusion proteins that carried genetic sequences encoding the MHC class II H-2^b^ restricted Eα peptide 52-68 (ASFEAQGALANIAVDKA), the OVA p323 peptide (ISQAVHAAHAEINEAGR), the MHC class I restricted OVA SIINFEKL peptide and the MHC class II H-2d-restricted influenza virus specific M2e peptide (ETPIRNEWGSR) were engineered and were equipped with Flag-tags. Corresponding fusion proteins with -the DD-dimer instead of the scFvCD103 were expressed with Flag-tags and purified as described before^77^. The purified material contained no endotoxin (0.1EU per mg of protein), it bound avidly to IgG in solid phase and CTA1 was enzymatically active as determined with the agmatine assay as described^77^.

### Adoptive cell transfers and chimeric mice

Splenic OT I or OT II T cells from CD45.1^+^ donor mice were labeled with Carboxyfluorescein succinimidyl ester (CFSE) (Life Technologies) and 1 × 10^6^ T lymphocytes were adoptively transferred into C57Bl/6 (CD45.2^+^) WT recipient mice. Immunizations were given on day 1 after transfer of cells. Bone marrow (BM) chimeras were made by i.v. transfer of 2 × 10^6^ BM donor cells from Batf3 and CD103-/- or WT mice into irradiated (1000 rad) recipient WT mice. BM reconstitution was achieved by 50/50% BM from Batf3−/− and CD103−/− or WT mice, respectively, so that chimeric mice would have cDC1 cells without (CD103−/−) or with CD103 (WT) expressing cells, while both strains would have cDC2 cells with CD103. Experiments with chimeric mice were initiated at 6 weeks after BM reconstitution.

### In vitro cell cultures

cDC–T cell co-cultures were established using highly enriched (>93%) cDCs from mLN taken from immunized or unimmunized WT mice. Briefly, highly enriched cDC1 cells were obtained by stepwise purification using MACS ;involving pan DCs selection (Miltenyi biotec) followed by positive selection with biotin tagged anti-XCR1 beads for maximum cDC1-enrichment (CD103^+^ CD11b^─^ DCs) (Stem cell technologies). Purified cDC1s (2 × 10^4^) were then co-cultured (1:10) with autologous, naive OT-II or Eα-TCR Tg CD4 T cells (2 × 10^5^) in 96 well U-bottom plates in IMDM medium containing 10% FCS (Sigma Aldrich), 1 mM sodium pyruvate (Gibco), 1 mM beta mercaptoethanol (Sigma) and 50 μg/ml Gentamycin (Gibco) and plated  at 2 × 10^5^ cells/well, in the presence or absence of ovalbumin (5μg/ml) or Eα-DD (1 μM) antigen and adjuvants; CTA1-II-CD103 (1 μM), Poly I:C (10 μg/ml), LPS (10 μg/ml) and CpG (2.5 μg/ml) for 48 h at 5% CO_2_ and 37 °C. T cell proliferation was determined by flow cytometry using the CFSE-dilution (Thermo Fisher Scientific) assay. Cytokine concentrations were determined by ELISA and intracellular staining for IFNγ used labeled anti-IFNγ Mab (BD Biosciences) and flow cytometry for detection.

Lymphocytes were isolated from the mLN or lung of immunized or immunized and challenged mice. Briefly, cells from the mLN were isolated by mechanical disruption and lung tissues were enzymatically digested using a kit from Miltenyi Biotec (Bergisch Gladbach, Germany) to obtain single-cell suspensions. Triplicate cultures were performed in 96-well plates (Nunc) and T cell recall responses were allowed 3 days in the presence of medium only or different concentrations of antigen, as indicated in the Figure legends. CD4 T-cell proliferation in vitro used CFSE-labeled OT-I or OT-II cells and cytokine determinations were performed on supernatants.

### Antibody and cytokine determinations

The ELISPOT assay was used to determine IL-17 or IFNγ producing cells from isolated mLN or lung lymphocytes. The dual kit for IL-17/IFNγ or single IFNγ spot forming cell (SFC) assessments was used according to the manufacturer’s instructions (Immuno Spot). Briefly, single cell suspensions of mononuclear cells were seeded in 96-well plates at 1 × 10^5^ cells/well and 1 μM of peptide was added and the plates were incubated for 24 h (5% CO_2_, 37 °C). Cytokine ELISPOTs were evaluated using a CTL ImmunoSpot analyzer. Cytokine determinations using ELISA were used for assessments of cytokines in culture supernatants. Prior to analysis samples were stored at −80 °C until further analyzed. Cytokine concentrations were determined in supernatants by ELISA (Duoset, R&D Systems, UK) and the concentrations of IFNγ, IL22, IL21, IL23, IL6, IL1β and IL-17A was done and given in pg/ml using a standard curve provided by the manufacturer.

MDCK and M2CK cells, which are MDCK cells that provide complementation of the M2 protein^41,42,78^, were seeded in a 96-well plate at 24,000 cells per well. 16 h later, the cells were blocked with 1% bovine serum albumin in PBS and incubated with serial dilutions of serum from individual mice. After washing with 0.05% Tween 20 in PBS buffer, binding was detected using a horseradish peroxidase-conjugated sheep anti-mouse IgG antibody (2000x GE Healthcare NA931V). After washing, TMB substrate (Tetramethylbenzidine, BD OptEIA) was added to every well. The reaction was stopped by addition of 1 M H_2_SO_4_, after which the absorbance at 450 nM was measured with an iMark Microplate Absorbance Reader (Bio Rad). This assay has been found to correlate well with functional assays of ADCC reactivity ^42^.

### Flow cytometry

Flow cytometry studies were performed as described in detail elsewhere. Briefly, dead cells were identified by 7-amino-actinomycin D or Live/Dead Aqua LIVE/DEAD Fixable Dead Cell Staining Kit (Life Technologies). Intracellular FACS stainings were achieved by the Fixation/Permeabilization Kit (eBioscience) according to the manufacturer’s instructions. Flow cytometry used a FACSAriaII or LSRII (BD Biosciences) and cells were analyzed using FlowJo X (Tree Star). Cell sorting of migratory cDC was done using a FACSAriaII (BD). The following fluorophores and conjugated antibodies were used for phenotypic analysis; M2e-specific tetramer-PE and control CLIP-PE and staining was performed at 37 °C for 30 min and followed by labeling on ice with cell surface specific markers for 30 min as follows; CD4 Alexa-700, CD8 AF647, CD3e BUV737, CD45.1 APCe Flour 780, CD69 PE-Cy7 and CD44 FITC. For labeling cDCs, we used CD11c BV421, MHC II (IA-E) Alexa-700, CD11b FITC, CD103 PE, and XCR1. APC AF647, CD19- APC-cy7 and F4/80 BV711 were used for a dump channel. Intracellular staining for detection of transcription factors; Tbet BV421, RORγt PE-CF594, GATA3 PE. T cell proliferation was assessed by CFSE-dilution which applied to both in vivo isolated cells as well as cells from in vitro cultures.

### In vivo cytotoxicity assay

Following transfer of 1 × 10^5^ OT I cells i.v. to autologous WT C57Bl/6 mice these were immunized i.n with 5 μM of the fusion proteins 12 h later. Splenocytes were taken from naïve mice and pulsed with SIINFEKL peptide (1 μg/mL peptide). Incubations were done at 4 °C for 1.5 h followed by 37 °C incubation for 30 min. The cells were labeled with CFSE (2 µM) (target cells) or unlabeled Far Red (2 µM)(bystander cells) without adding peptide (negative control); 10 × 10^6^ CFSE and 10 × 10^6^ Far Red labeled splenocytes (20 × 10^6^ cell transfer /mouse) were mixed and transferred to autologous WT recipient mice. After 20 h, mLN and spleens of immunized mice were stained with Live dead aqua dye and analyzed by flow cytometry. Cytotoxicity was then determined by calculating the % killing as a consequence of $\left( {1 - \left( {\frac{{\% CFSE}}{{\% Far\,Red}}} \right)} \right) \times 100$.

### Single cell RNAseq and Seurat and Monocle 3 analysis

Migratory cDCs (MHC II ^high^ CDllc ^high^) from naïve/PBS-treated or i.n fusion protein immunized mice were isolated from mLN from a pool of 7–8 C57Bl/6 mice and FACS -sorted on an Aria II cell sorter (BD Bioscience). cDCs exposed to the fusion proteins were identified on the basis of their expression of the labeled Eα-peptide+ MHC classII -specific Yae Mab. Labeling of cells prior to sorting by flowcytometry (Fusion, BD) included the following specific Mabs Yae, anti-CD4, anti-CD8, anti-B220 and anti-F4/80, as described^5^. Cells were sorted into IMDM, before being pelleted and resuspended in IMDM-10% FBS. Approximately 5000 sorted celles were loaded on the 10x Chromium Controller (10x Genomics). The scRNA-seq libraries were prepared following the user guide manual provided by the 10X Genomics company. Data from scRNA-seq of DC sorted samples were individually processed using **c**ell **r**anger **a**nalysis **p**ipeline (10X genomics platform) and reads aligned on the mm10 genome assembly. The output form Cell Ranger then further analyzed with Seurat Seurat 3.0. Raw UMI count matrices were loaded and merged into a single Seurat object. Cells were discarded if they met any of the following; percentage of mitochondrial counts greater than 6% per cell, number of unique features either below 200 or above 3500, T cell genes (*‘Cd3d’, ‘Cd3e’, ‘Cd3g’, ‘Trac’, ‘Trbc1’, ‘Trbc2’*) greater than 0.1 percent and B cell genes (*‘Cd79a’, ‘Cd79b’, ‘Ms4a1’, ‘Cd19’*), greater than 0.1 percent. Gene counts were log normalized and mean centered and scaled by their standard deviation and the following variables regressed out: the number of UMI and the percentage of mitochondrial counts. Finally, cells were clustered and UMAP applied to further reduce dimensionality for visualization. Differential gene expression was performed using the FindAllMarker function with Wilcoxon Rank Sum test and minimum of 0.25 log fold-change. For analysis using Monocle v3, the Seurat object was imported using the as.cell_data_set function from SeuratWrappers and further processed. Pseudotime analysis was performed using standard settings and with root node selection in cluster 0. Modules of co-regulated genes, differentially expressed across pseudotime, were identified using the function find_gene_modules using a list of Louvain resolution and selecting the one with highest value.

### Gene set enrichment analysis (GSEA)

For GSEA analysis, differentially expressed genes for CTA1 (DD- and CD103) versus PBS in cDC1 population were calculated using the Wilcoxon rank sum test via the wilcoxauc function of the presto package using default parameters (including Benjamini-Hochberg false discovery rate correction) and filtered on logFC > 1 and padj < 0.05. GSEA was run on pre-ranked genes using the fgsea package (Korotkevich, G., V. Sukhov, and A. Sergushichev. 2019. Fast gene set enrichment analysis. bioRxiv 060012). For each enrichment graph we report p, padj (FDR q) and NES (enrichment score normalized to mean enrichment of random samples of the same size) values in the figure.

## Supplemenatry information

Author Checklist
Supplemenatry figures
Supplemenatry table
