# Supplementary material for: ADP-ribosylating adjuvant reveals plasticity in cDC1 cells that drive mucosal Th17 cell development and protection against influenza virus infection
Source: Mucosal Immunol. 2022 Apr 13;15(4):745–61. doi: 10.1038/s41385-022-00510-1 (PMC9259495; doi:10.1038/s41385-022-00510-1)
Supplement: Supplementary file 2 — Supplemenatry figures [file 41385_2022_510_MOESM2_ESM.pdf]

Supplementary Fig 1

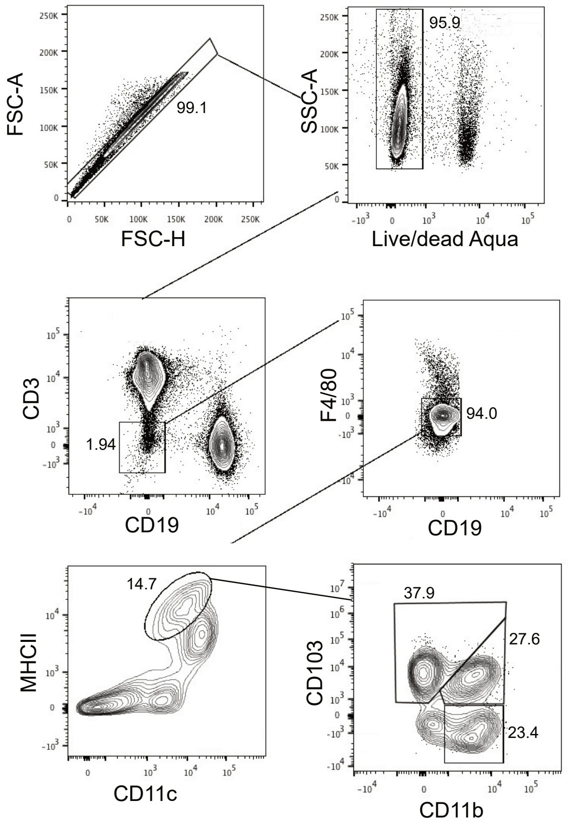

Supplementary Fig 2

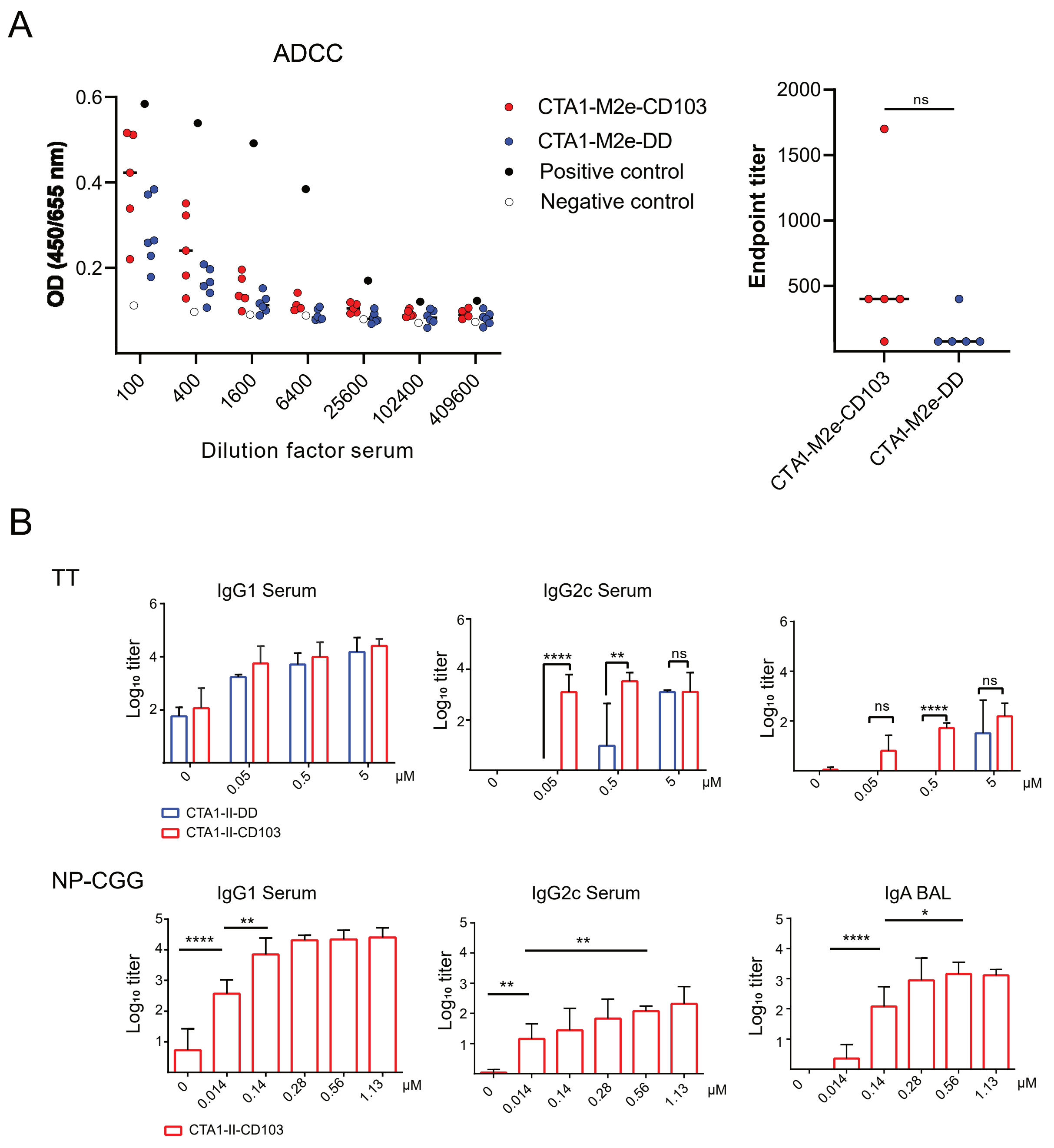

Supplementary Fig 3

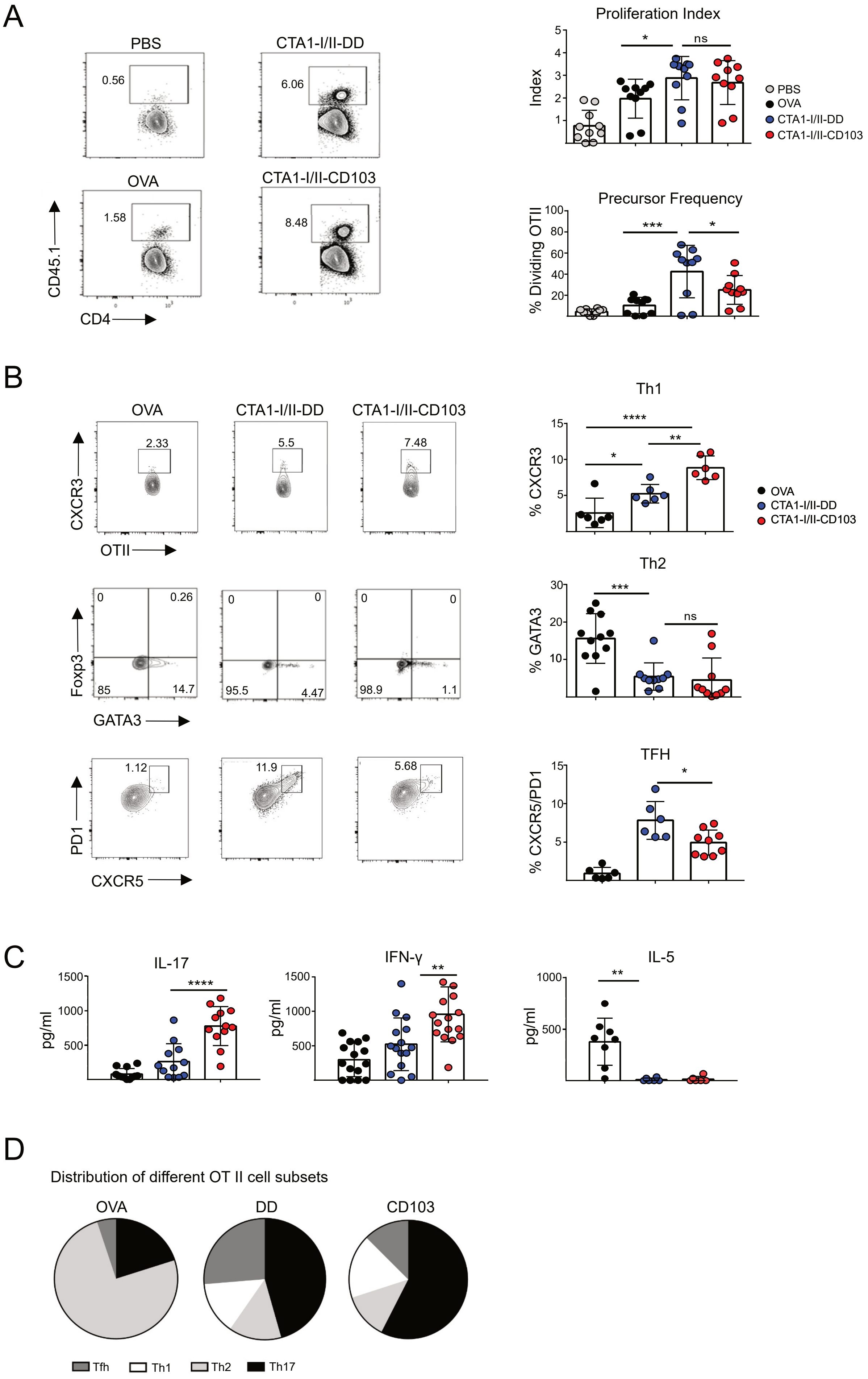

Supplementary Fig 4

A

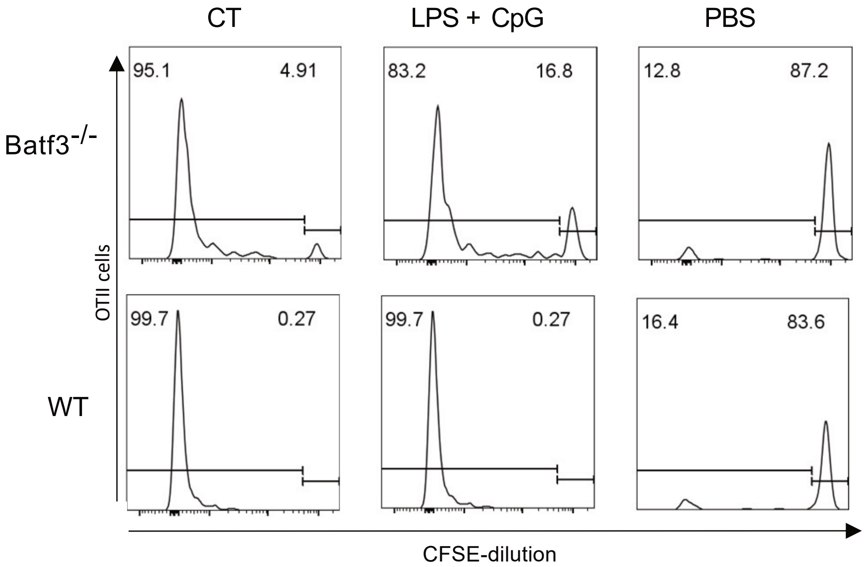

B

In vitro experiment

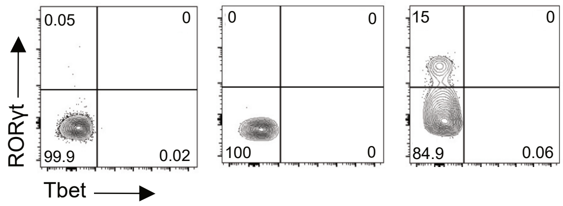

cDC1 cells	+	-	+
OTII CD4 T cells	+	+	+
CTA1-II-CD103	-	+	+

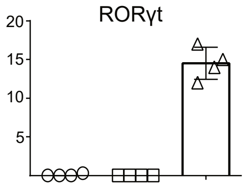

cDC1 cells	+	-	+
OTII CD4 T cells	+	+	+
CTA1-II-CD103	-	+	+

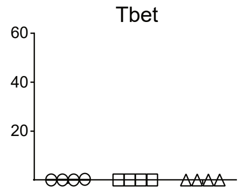

cDC1 cells	+	-	+
OTII CD4 T cells	+	+	+
CTA1-II-CD103	-	+	+

Supplementary Fig 5

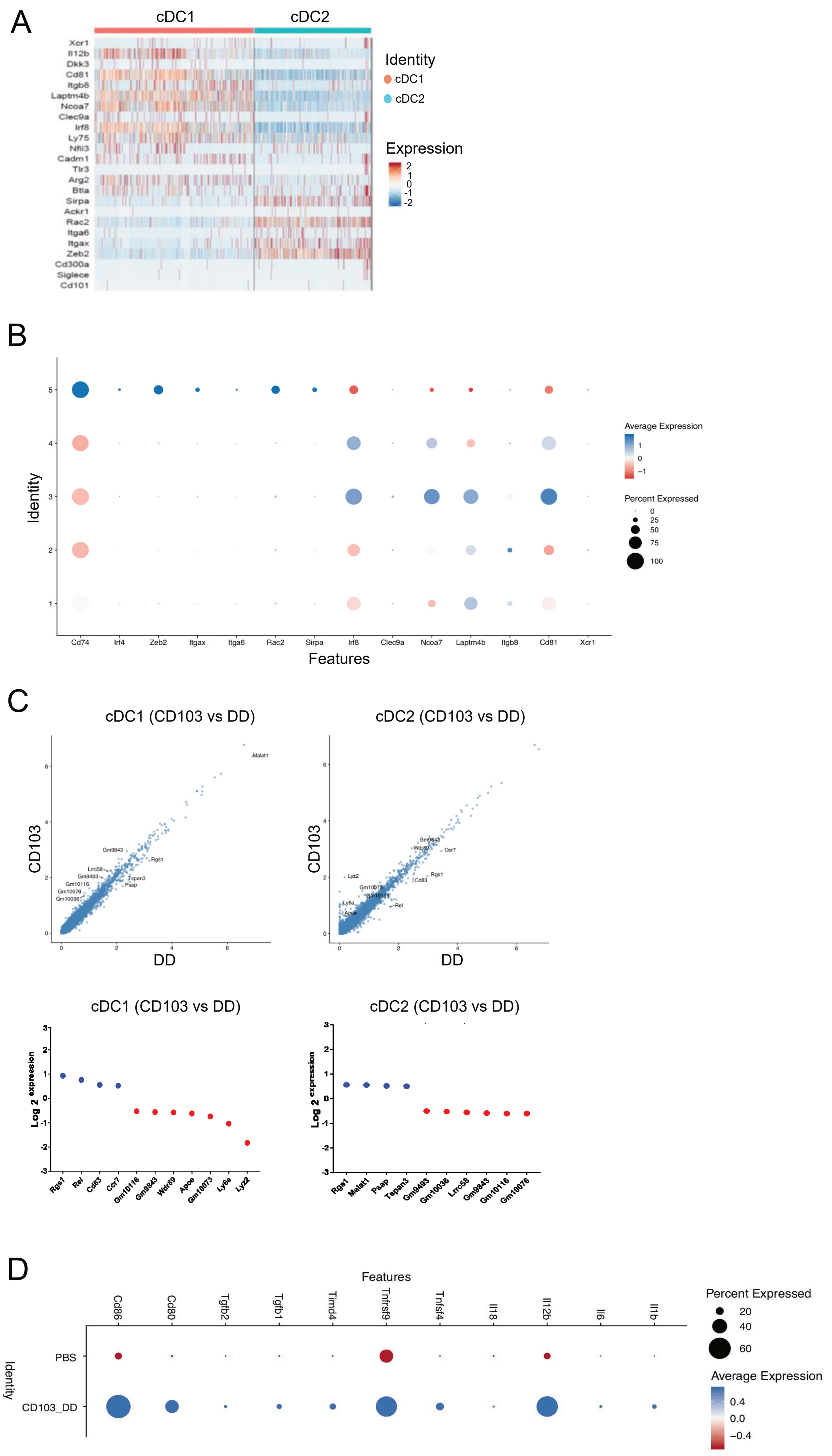

Supplementary Figure Legends

Supplementary Figure 1:

Gating strategy

Flow cytometry gating strategy for defining cDC subsets in freshly isolated mesenteric lymph node (MLN) cells.

Supplementary Figure 2:

The CTA1-CD103 is superior to the CTA1-DD adjuvant at stimulating antibody responses to admixed proteins

Functional assessment of the anti-M2e-response in serum following i.n immunizations with CTA1-M2e-CD103 or CTA1-M2e-DD. MDCK and M2CK cells, which are MDCK cells that provide complementation of the M2 protein (40, 41, 77), were incubated with serial dilutions of serum and binding was detected using HRP-conjugated anti-mouse IgG antibody and the reaction was read at 450 nM using an absorbance reader. The assay correlates well with functional assays of ADCC reactivity (41) Values are given as individual values and mean endpoint titers of 5 mice in each group (A). Mice were immunized with a fixed dose (5 μg) of admixed NP-CGG or TT antigen and a range of doses of the CTA1-II-CD103 or CTA1-II-DD adjuvants were given i.n in 3 doses with 10 days apart. The immune responses were determined 8 days after the final immunization in serum and bronchioalveolar lavage (BAL) and values are given as mean $\log_{10}$-titers ±SD of 5 mice in each group and one representative experiment of three is shown (B). Statistical significance was calculated using ANOVA with Dunnett's post-test; p-values ** $p<0.01$, *** $p<0.001$, **** $p<0.0001$.

Supplementary Figure 3:

CTA1-II-CD103 stimulated CD4 T cell subset differentiation dominated by Th17 cells

Wild-type C57Bl/6 mice ($CD45.2^+$) were given CFSE-labeled $CD45.1^+$ OT-II transgenic T cells by adoptive transfer and subsequently immunized i.n. with equimolar doses of 5μM of OVA, CTA1-II-DD, or CTA1-II-CD103 on the next day. OT II cells from mLN were analyzed on day 4 for in vivo proliferation by assessing CFSE-dilution by flow cytometry (A). Differentiation into the different functional subsets was done by phenotypic analysis using labelled antibodies to CXCR5 and PD-1 for Tfh, GATA3 for Th2, Tbx21 and CXCR3 for Th1, Foxp3 for Tregs. Gating was done using FMO to identify the subsets. Distribution of OT II cells into the different subsets was are given as mean % ±SD of all OT II cells, as indicated, and these are representative of three independent experiments with 2-6 mice in each group (B). Lymphocytes from the mLN from immunized mice as in A were cultured in triplicates with recall antigen p323 peptide for 96 h, after which supernatants were collected and assessed for cytokine content, as indicated. Cytokine production is given in pg/ml ±SD for each group (C). Statistical significance was calculated using analysis of variance (ANOVA) with Dunnett's post-test. * $p<0.05$, ** $p<0.01$, *** $p<0.001$, **** $p<0.0001$. Distribution of the different CD4 T cell subsets of OT II cells following a single i.n immunization as indicated. Values are given as a pie chart representing mean % of all OT II cells and these are calculated from three independent experiments with 2-6 mice in each group (D).

Supplementary Figure 4:

CTA1-II-CD103 acting on cDC1 cells *in vitro* promotes Th17 differentiation

Following adoptive transfer of CFSE-labelled naïve OT II T cells into WT or Batf3-/- mice we isolated mLN on day 4 and Ly5.2+ T cells were analyzed for in vivo proliferation following i.n immunizations with OVA (5 μg) given together with CT(1 μg) or LPS + CpG (10 μg) adjuvants. Histograms showing representative examples of OT II cell proliferation in mLN in response to immunizations (A). Highly enriched cDC1 cells from unimmunized mice were cultured together with naïve CFSE-labelled OT-II cells (CD45.1+) in a 1/10 ratio and 5μM of CTA1-II-CD103 fusion protein was added to these cultures as indicated. The frequency of Rorγt^{+} or Tbet^{+} OT-II cells in triplicate cultures was determined on day 3 by flow cytometry using labeled antibodies and mean values±SD from one representative experiment of three giving similar results (B).

Supplementary Figure 5:

Single-cell RNAseq analysis of cDCs from mLN

Gene heat map (A) and gene dot plot (B) for differentially expressed cDC1 and cDC2 associated gene sets for migratory cDC cells. Plot illustrating the gene sequence expression pattern in CD103 vs DD-constructs in cDC1 and cDC2 cells, respectively(C upper panels). Top 20 differentially expressed genes (DEG) in CD103- compared to DD-targeted cDC1 and cDC2 cells analyzed 20h following an i.n administration of 50 μM doses and analyzed, as indicated (C lower panels). Gene signatures identifying Th17 promoting gene functions in cDC1 cells isolated from CTA1-DD/CTA1-CD103 vs PBS-treated mice (D).
